# Non-invasive sampling of bats reflects their potential as ecological indicators of elemental exposure in a diamond mining area, northern Limpopo Province, South Africa

**DOI:** 10.1007/s11356-021-16466-x

**Published:** 2021-09-30

**Authors:** Dawn Cory-Toussaint, Peter J. Taylor, Irene E. J. Barnhoorn

**Affiliations:** 1 Department of Zoology, School of Natural and Mathematical Sciences, University of Venda, P. Bag X5050, Thohoyandou, 0950 Republic of South Africa; 2 Department of Zoology and Entomology, Afromontane Unit, University of the Free State – QwaQwa Campus, P. Bag X13, Phuthaditjhaba, 9866 South Africa

**Keywords:** Bioaccumulation, Heavy metals, Chiroptera, Ecotoxicology, Biomarkers, Opencast mining

## Abstract

Bats have been proposed as reliable bioindicators for monitoring bioaccumulation of elements and chemicals in natural and transformed ecosystems. Non-invasive methods are becoming more popular as research moves away from destructive methodologies. We present the first concentrations of 23 elements in *Mops condylurus* and *Tadarida aegyptiaca* (Molossidae) fur and blood from an opencast diamond mine and reference area using inductively coupled plasma mass spectrometry (ICP-MS). Concentrations of B, K, Rb and Cd in the bats’ fur were significantly higher in the mining footprint compared to the reference area (*P* < 0.05). Other elements such as Zn and Hg were significantly higher in the blood of bats from the mining footprint than the reference area (*P* < 0.05), whereas Mn was significantly higher in the blood of bats from the reference area than from the mining footprint (*P* < 0.05). Sixteen of the 22 elements above the limit of detection, with the exception of Ba, were significantly higher in the fur samples than in the blood due to elements being incorporated over time into the fur as it grows, whereas blood reveals short-term exposure to elements. Concentrations of most of the elements were reasonably low except Al, Fe and Zn. In general, the element concentrations particularly in the fur samples were comparable with other international studies reporting elemental fur concentrations from anthropogenically impacted and natural areas. Fur and blood have the potential to be viable indicators of environmental toxicity, but research is required on toxic thresholds and physiological and ecological unknowns around element concentrations in bat tissues and organs.

**Supplementary Information:**

The online version contains supplementary material available at 10.1007/s11356-021-16466-x.

## Introduction

Heavy metals and trace elements are naturally occurring minerals present in the earth’s crust, which under natural conditions, leach into water from the surrounding rock and soil, and are taken up by plants and animals (Garrett 2000; Nagajyoti et al. 2010; Flache et al. 2015). The general accepted definitions of heavy metals based on the density and/or specific gravity of elements are “hopelessly imprecise, leads to confusion and is useless to describe toxic properties” (Smith and Norberg 2015). We consider three main groups of elements pertinent to our study in line with Smith and Norberg (2015): main metal groups, transition metals and metalloids. Based on the confusion and multiple re-definitions of heavy metals, from this point forward, we will simply refer to heavy metals, trace elements, macronutrients and micronutrients using the term “elements”.

Element bioaccumulation, toxicity and resulting effects have been a subject of interest for many years (Jakimska et al. 2011; Bat et al. 2020). The effects and impacts of elements and other pollutants that bats have been and are currently exposed to is gaining increased attention (Zocche et al. 2010; Griffiths et al. 2014; Lovett and McBee 2015; Naidoo et al. 2016; Carrasco-Rueda et al. 2020). Effects of element pollution include but are not limited to changes relating to bat diversity, alteration of relative abundances, population structure changes, negative impacts on flight activity, disruption of plasma glucocorticoids, central nervous system alterations (causing a general lack of coordination, loss of movement, tremors, paralysis), damage to internal organs (renal inclusion bodies), hemochromatosis (“iron overload”), DNA damage, immunosuppression and mortality (Zocche et al. 2010; Karouna-Renier et al. 2014; Zukal et al. 2015; Naidoo et al. 2015, 2016; Mina et al. 2019). However, the concentration at which elements are toxic and or fatal to bats is unknown.

Persistent environmental pollutants are an underrated threat to bats and the manner in which contaminants transfer, bio-magnify through trophic levels and accumulate within an organism (in tissues and organs) is fairly complex (Clark et al. 1986; Flache et al. 2018; Mina et al. 2019). Different bat species may show specific element concentrations in their tissues and organs associated with variations in exposure within different foraging habitats, dietary guilds and physiological regulation of elements (Karouna-Renier et al. 2014; Zukal et al. 2015; Flache et al. 2015; Hernout et al. 2016a; Becker et al. 2018; Flache et al. 2018; Moreno-Brush et al. 2018; Carrasco-Rueda et al. 2020; de Souza et al. 2020). There could be numerous instances where high levels of elements may not be due to the contamination of the environment, but may be an artefact of the bat’s diet. For example, *Myotis myotis* has been reported to contain high levels of Mn that may come from their predominant carabid beetle diet which reportedly strengthen their mandibles with Mn (Flache et al. 2015). It is evident from the literature that different species of bats are exposed to different types of contaminants based on their dietary guild, sex, age and seasonality (Clark et al. 1986; Naidoo et al. 2013; Hernout et al. 2016a; de Souza et al. 2020). However, this differs depending on the element and possibly species for example; no differences in concentrations of mercury in the fur of *Carollia perspicillata* and *Phyllostomus elongatus* were found to be attributed to sex and age, which indicated that Hg was not accumulated over time (Moreno-Brush et al. 2018).

Using fur could be a good biomarker as the roots are in contact with the bloodstream, and thus metals may be incorporated into the fur during growth and additionally fur also stores external airborne particles, thus external exposure and ingestion of elements could be investigated (de Souza et al. 2020). Fur in comparison to blood provides an indication of a longer time exposure to elements and provides information concerning the exposure of an animal at the time of the tissue formation (Fraser et al. 2013; Hernout et al. 2016a). For example, Flache et al. (2015) used bat fur to monitor bat’s exposure to potentially toxic metals in their foraging habitat and reported trace metal concentrations of Cd, Cu, Mn, Pb and Zn in fur samples collected from *M. bechsteinii*, *M. daubentonii*, *M. myotis* and *Pipistrellus pipistrellus.*

Metal concentration in fur varies at different times of the year; e.g. fur collected prior to the annual moult cycle may contain higher metal concentrations than those during or after the moult; therefore, the moult cycle must be taken into consideration when collecting samples (Fraser et al. 2013; Flache et al. 2015; Hernout et al. 2016b). The moult cycles of bats have not been well studied (particularly in South Africa) and Fraser et al. (2013) provided a summary of the moult cycles of ~27 bat species and highlighted that timing and pattern of the moult differed between species, sex, reproductive status and age. Blood, on the other hand, provides information concerning a more recent exposure as it is gradually replaced (Fraser et al. 2013). Powolny et al. (2019) showed that in wood mice (*Apodemus sylvaticus*) sampled along a pollution gradient, blood could be a good indication of internal organ levels of Se, Pb and thallium (Tl). On the contrary, blood concentrations of titanium (Ti), Cd, Fe, Cu, Mo and Zn were not good indicators of internal organ concentrations (Powolny et al. 2019).

Being the second highest species-rich mammalian order in the world representing approximately 20% of global mammal diversity, bats form a large component of global biodiversity and deliver key services to both ecosystems and humans (pest control, pollination, seed dispersal and forest regeneration) (Jones et al. 2009; Kasso and Balakrishnan 2013; Bayat et al. 2014; Riccucci and Lanza 2014; Taylor et al. 2018). Bats are considered good potential biodiversity, environmental and ecological indicators due to their small size, high mobility, high metabolic rates and associated high prey intake of between 40 and 100% of their body weight each night, global distribution and coexistence with humans thus increasing their exposure to a range of contaminants (Hickey et al. 2001; Jones et al. 2009; Russo and Jones 2015; Zukal et al. 2015).

We investigated the elemental concentrations in blood and fur of two species of open-air foragers; *Mops condylurus* (Angolan free-tailed bat; A. Smith 1833) and *Tadarida aegyptiaca* (Egyptian free-tail bat; É. Geoffroy Saint-Hilaire 1818) collected during summer from an opencast diamond mine and reference site in northern Limpopo Province. Our aims were to (a) compare the concentrations of elements in bat fur and blood between the mining footprint and reference area, (b) determine if there were correlations between fur and blood element concentrations, (c) investigate possible ingestion of elements through prey available and consumed by individual bats and (d) how the element concentrations compare with those reported in the literature. The concentrations of elements in blood and fur could provide insight into using bats as bioindicators for environmental change (for example water and/or prey quality (Jones et al. 2009)). We expect that bioaccumulation of elements in bats fur and blood is significantly higher over the mining footprint than the adjacent reference area. Opencast mining operations could provide a source of high element exposure through the liberation and increased availability of elements in large waste water (or tailings) dams from the processing of diamond containing rock (kimberlite). Alternatively, we may find no difference in heavy metal concentration in the fur and blood of bats active over the opencast mine and adjacent reference area. Correlations between element concentrations between fur and blood could be a reliable indicator of internal element concentrations. Faecal pellet analysis and available prey items (relative abundance) may indicate a potential pathway of elemental ingestion during the time of the study particularly if the prey had an aquatic life-stage.

## Materials and methods

### Study site

**Fig. 1:**
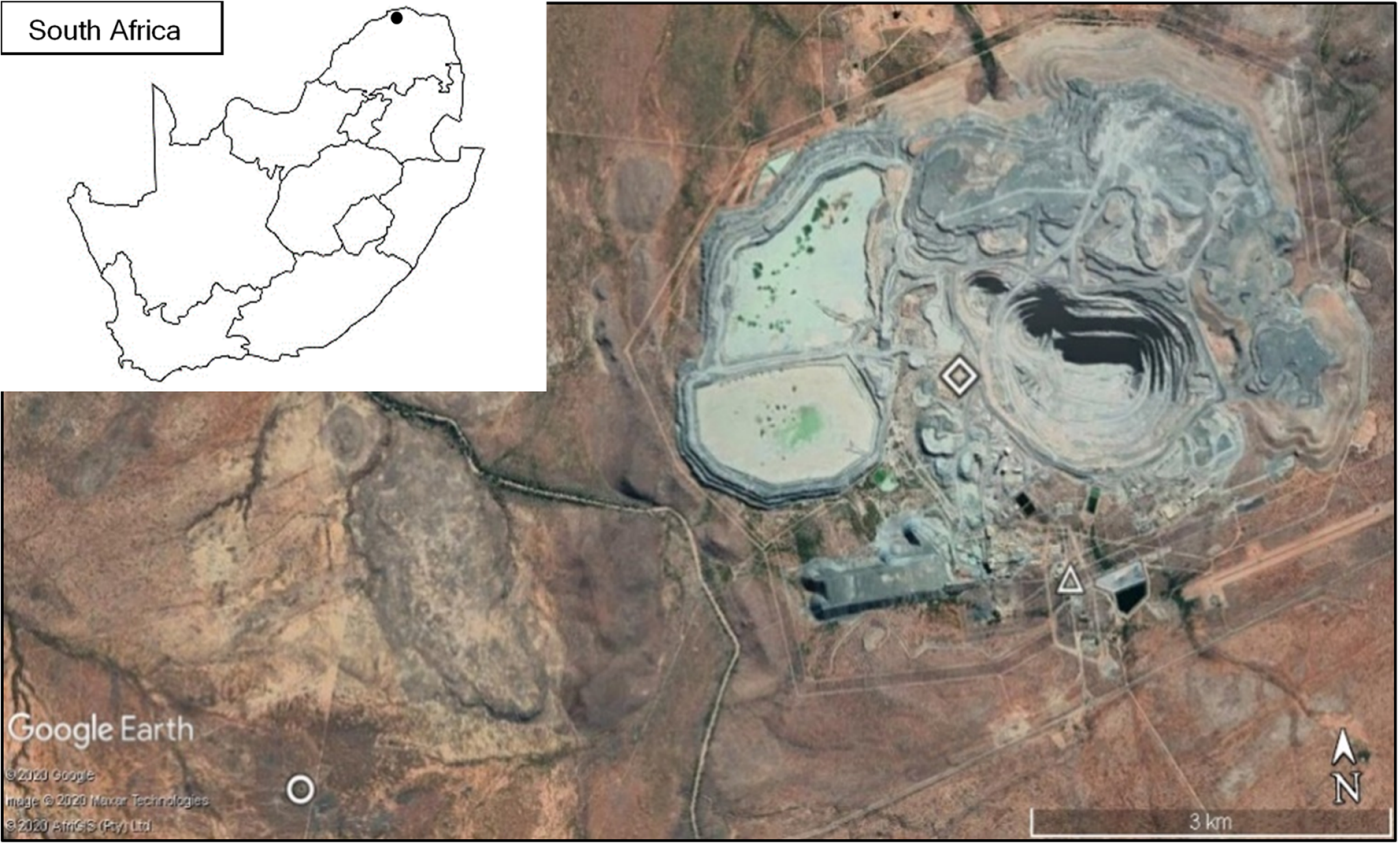
The layout of the study site and capture sites: the Venetia Diamond Mine (diamond shape) footprint and associate capture site (white triangle) and the reference area capture site at a temporary water pan (open circle) on Corea Game Farm. The solid fill dot in the insert map of South Africa indicates the approximate location of the Venetia Diamond Mine in northern Limpopo Province

The study was conducted on the De Beers Venetia Diamond Mine, in the Limpopo Province (−22.449593° S, 29.319494° E) and Corea Game Farm (−22.462280°S, 29.256442°E) (Fig. 1). The Venetia Diamond Mine has been in operation since 1992 (https://www.debeersgroup.com/the-group/our-history) in the Limpopo mobile belt, where a complex kimberlite pipe containing diamonds is situated (Brown et al. 2009). A temporary water pan on Corea Game Farm was used as the reference area that was situated ~5km in a straight line from the large wastewater dam on the western side of the mining footprint and ~6.5km from the bat roosting site (Fig. 1). Corea Game Farm is situated within the diverse Musina Mopane Bushveld vegetation unit dominated by mopane trees (*Colophospermum mopane*) on poorly developed soils (Mucina and Rutherford 2011). The mine footprint is situated in the Limpopo Ridge Bushveld, which is also dominated by mopane trees. The climate of the area is characterised by very dry winters and hot summers with mean annual precipitation between 300 and 400mm (Mucina and Rutherford 2011).

### Bat capture

Bat capture was conducted during December 2018 (summer) on the Venetia Diamond Mine and on Corea Game Farm (reference area). All captured bats were initially held in cotton bags, processed and identified to species level. Free standing nylon monofilament mist nets (ECOTONE, Gdynia, Pomerania, Poland) and an Austbank harp trap (Faunatech, Australia) were used to capture bats. On the mine, free standing mist nets of 9 m and 12 m were extended parallel to the edges of the waste water dams. Due to the total illumination of the active mining footprint, the tempestuous summer weather and resulting billowing motion of the free-standing mist nets, capture success and sampling effort (8 h and 45 min) in the mist nets was poor on the mine. Three individual bats (two bat species from the family Vespertilionidae and a single Rhinolophid) were captured. An Austbat two-bank harp trap was placed at the entrance/exit of a known roost of free-tailed bats (Molossidae) in an unused building on the Venetia Diamond Mine to capture individuals from the roost. The harp trap was deployed for 22 h and 40 min with a resulting bat capture success of 26 individuals of which 24 were *M. condylurus* and two were *Chaerephon pumilus* (Little free-tail bats; Cretzchmar 1826). Trapping on the reference area consisted of two sets of 9 m and 12 m mist nets strategically placed across temporary water pans. Two mist nets were placed one above the other to have a resulting drop of ~5 m. The mist nets were open for 8 h, and the harp trap was deployed for 25 h and 20 min and yielded 22 individuals of which 10 *T. aegyptiaca* and two *M. condylurus* were captured. The remaining 10 individuals belonged to the family Vespertilionidae and were not analysed in the current study as there were no comparative individuals from the mine footprint. Overall, female bats (*n* = 32) dominated over males (*n* = 19) during the active capture sessions particularly over the reference area (females, *n* = 16; males, *n* = 6). Four adult males (mining footprint) and seven non-lactating adult females (mining footprint: *n* = 2; reference area: *n* = 5) were selected from the captured individuals. See supplementary information (SI) Table S1 for selected individual’s sex, reproductive state and morphological measurements. It would have been ideal to only have males representing the sample as in some instances, sex and reproductive status effects element concentrations in some bat species; e.g. females eliminate metals better than males through lactation (Hernout et al. 2016a) which is why only non-lactating adult females were selected.

### Fur and blood collection

Since the collection period was during December, we assume that the bats had already had their annual summer moult (Fraser et al. 2013). Fur was collected using a pair of sharp surgical scissors (Lasec, Laboratory & Scientific Equipment Company (pty) Ltd.). Fur was carefully clipped from the dorsal side of the bat, as close to the skin as possible, starting from the pelvic region and ending between the scapulae. Individual fur samples were immediately placed into small zip-lock bags and marked.

Blood was only taken from males and reproductively inactive females, not pregnant nor lactating. Blood samples were taken from each bat in line with Smith et al. (2010). Each bat was gently restrained in the left hand. The left wing was carefully extended to expose the inner upper arm which was then sanitised using an alcohol swab. Slight pressure was applied to the brachial vein near the base of the upper arm, and the tip of a sterile 24-gauge needle was used to puncture the vein. The resulting bead of blood was sampled using 125-μL heparinised Clinitubes (Radiometer, Denmark). Acceptable volumes of blood between 62.5 and 120 μL were taken (2.9 μL g^−1^– 5.5 μL g^−1^, respectively) (Smith et al. 2010). A small butane torch (Zengaz, ZT-50) was used to melt the ends of the capillary tubes, and with a quick twist of the melted glass, the ends were sealed. The blood samples were immediately refrigerated.

Fieldwork was conducted with approval of the animal ethics clearance by Research and Innovation, Office of the Director, University of Venda, Project No: SMNS/19/ZOO/02/0307. All bats sampled survived and were successfully released at their respective sites of capture. Bats captured on Corea Game Farm were processed the same night of capture and immediately released. Only the bats captured from the Venetia Diamond Mine using the harp trap were held for ~12 h due to mine security access and were offered mealworms and water prior to release back on the mine the following evening.

### Element concentrations in fur and blood by ICP-MS

Fur (*n* = 11) and blood (*n* = 11) samples were tested for elements antimony (Sb), aluminium (Al), arsenic (As), barium (Ba), boron (B), calcium (Ca), cadmium (Cd), chromium (Cr), cobalt (Co), copper (Cu), iron (Fe), lead (Pb), manganese (Mn), mercury (Hg), molybdenum (Mo), nickel (Ni), potassium (K), rubidium (Rb), selenium (Se), strontium (Sr), tin (Sn), vanadium (V) and zinc (Zn) (Table 1). The elemental analysis was conducted by the Central Analytical Facility (CAF), University of Stellenbosch, Western Cape, using an Agilent 7900 quadrupole inductively coupled plasma-mass spectrometer (ICP-MS) with a High Matrix Introduction (HMI) system. We acknowledge that the absence of washing the fur samples prior to analysis could be a potential source of error and renders comparisons with available literature a challenge. Biological samples were weighed directly into 15-ml acid-cleaned Falcon® tubes. And 0.25 ml ultra-pure nitric acid and 0.25 ml ultra-pure hydrogen peroxide were added to each tube. The tubes were then placed in an oven at 60 °C for 30 min. After samples were digested, 2 ml of ultra-pure de-ionised water (18 MΩcm^−1^, Milli-Q® IQ Element, Merck KGaA, Darmstadt, Germany) was added.

**Table 1:** The classification (indicated by “X”) of elements tested for in the blood and fur of bats based on Smith and Norberg (2015). Elements marked with “*” indicate their presence in animal and plant cells but their biological importance is largely unknown (see Bánfalvi 2011)

Element	Metalloid	Main group metal	Transition metal
Antimony (Sb)	X		
Aluminium (Al)*		X	
Arsenic (As)*	X		
Barium (Ba)*		X	
Boron (B)	X		
Calcium (Ca)		X	
Cadmium (Cd)*			X
Chromium (Cr)			X
Cobalt (Co)			X
Copper (Cu)			X
Iron (Fe)			X
Lead (Pb)*		X	
Manganese (Mn)			X
Mercury (Hg)*			X
Molybdenum (Mo)			X
Nickel (Ni)			X
Potassium (K)		X	
Rubidium (Rb)*		X	
Selenium (Se)	X		
Strontium (Sr)*		X	
Tin (Sn)		X	
Vanadium (V)			X
Zinc (Zn)			X

Each sample was introduced through a ~0.2ml min^−1^ concentric nebulizer into a Peltier cooled spray chamber. Before the sample was introduced into the high temperature plasma, argon dilution gas from the HMI configuration was added. Helium and H_2_ gas were used respectively as collision and reaction gases in a 4th generation Octopole Reaction System (ORS) to remove polyatomic interferences from the analytes of interest. Boron, Al, V, Cr, Mn, Fe, Co, Ni, Cu, Zn, As, Sr, Mo, Cd, Sn, Sb, Ba, Hg and Pb were measured in He collision mode, while H_2_ reaction gas was used for Se. The Agilent 7900 instrument was optimized daily for sensitivity and low oxide ratios (CeO/Ce < 0.3%).

Concentrations of elements in fur and blood were reported as μg g^−1^ and μg ml^−1^ wet weight (w.w.) and for the statistical analysis, were converted to parts per million (ppm). For comparative purposes, reported dry weight element concentrations in the literature were converted to wet weight by dividing the values by four (see Hernout et al. 2016b; Ferrante et al. 2018) and are presented in Supplementary information Table S2.

### Calibration and method validation

The National Institute of Standards and Technology (NIST) traceable multi-element stock solutions (INORGANIC VENTURES – 300 Technology Drive, Christiansburg VA 24073) were used to prepare instrument calibration standards in 2% HNO_3_ ranging from 1–1000 ppb. Single element Hg standards ranging from 0.5–5 ppb were prepared in 2% HNO_3_ + 2% HCl. Suprapur (65%) double distilled nitric acid (HNO_3_) and Suprapur (30%) hydrochloric acid (HCl) were purchased from Merck KGaA, Darmstadt, Germany.

Initial calibration verification standards to confirm accuracy and calibration linearity was analysed directly after instrument calibration. NIST traceable multi-element standards and single element Hg standard from De Bruyn Spectroscopic Solutions, Bryanston, South Africa, was used for this purpose.

Three replicates of Seronorm L2 (blood) were used as a reference material for the samples in the current study to evaluate if the selected digestion methods were efficient in collecting the extractable mineral content from the samples and can be accurately and reproducibly measured by ICP-MS. The resulting calibration curve and R^2^ value of the response of the Agilent 7900 ICP-MS using three replicates of Seronorm L2 was y = 0.933x + 5.330 and 0.9985, respectively.

Instrument drift and matrix effects were monitored and corrected by internal standard elements (^45^Sc, ^89^Y, ^115^In, ^72^Ge, ^103^Rh) added automatically from a multi-element mixture in 2% HNO_3_ to each sample and standard before introduction into the ICP-MS instrument.

### Faecal pellet preparation and insect reference sampling

Each cotton holding bag was inspected and faecal pellets were collected in order to identify if captured bats were consuming insect orders that had an aquatic life-cycle phase. Faecal pellets were softened using 98% ethanol and spread between two slides. Arthropod remains in the faecal pellets were identified to order and where possible family using the arthropod key in Kunz and Parsons (2009) and the study reference samples identified using Picker et al. (2004). Percentage frequency for each arthropod order present in the faecal pellets was visually estimated in accordance with Kunz and Whitaker (1983).

A light trap consisting of an 11 watt (600 lum) warm-white light bulb suspended over a container of water and powered by a portable power unit (EcoBoxx Qube 160, South Africa) was used to collect a representative sample of arthropods available to bats foraging over the mining footprint and over the temporary water pan on the reference area. The light trap was regularly checked and insects were collected. Insects were sorted predominantly to order level and where possible to family or species. Representative individuals of each order/family were crushed into fine pieces with a pestle to simulate the grinding action of a bat’s teeth and mounted between two microscope slides (Lasec Laboratory & Scientific Equipment Company (pty) Ltd.). Each insect order was weighed (g, w.w.) to calculate relative abundance to determine what was available to foraging bats during the sample period.

The faecal pellets and insect remains were inspected and photographed using a Zeiss Stemi 508 microscope (Karl Zeiss, Germany) fitted with a 4mp Axiocam ERc 5s (Rev. 2.0) camera.

### Statistical analysis

Data was analysed in R (Version 1.1.456, RStudio, Inc.). Results of element concentrations in fur and blood are presented as median and range as the values in many instances varied considerably. *T*-tests assuming equal variances were used to test if there was a significant morphological difference between *M. condylurus* and *T. aegyptiaca* that may affect the results of the element analysis.

Each element concentration data set of the bat fur and blood were tested individually for normality using Shapiro tests (*P* < 0.05 not-normally distributed and *P* > 0.05 normal distribution) to make comparisons between the mining footprint and reference site, and between the total fur and blood samples. Where data were not normally distributed, Levene’s tests were used to determine the homogeneity of variances. Nonparametric Mann-Whitney *U*-tests were used where data did not have a normal distribution and equal variances. Where two data sets that were compared differed in their distribution, a two-sample t-test assuming equal or unequal variances was used based on the results from Levene’s tests. ANOVAs (ANOVA (AOV)) were run where data had a normal distribution. Wilcoxon matched paired tests were used to compare fur and blood concentrations of elements where data did not have a normal distribution.

Spearman’s correlation coefficient in R (cor.test) was used to determine if there were any correlations between the element concentrations in blood and fur.

## Results

### Trace elements by ICP-MS

*Tadarida aegyptiaca* and *M. condylurus* belong to the family Molossidae and are similar from an ecological and morphological perspective (masses and forearm lengths not significantly different; *P* = 0.30 and *P* = 0.21 respectively); thus, we do not expect a phylogenetic effect of heavy metal and trace element concentrations of the fur and blood although we acknowledge that this could be a limitation. Table 2 indicates the medians, range and LOQ values of each element. Elements Al, Cd, Co, Cr, Mo, Ni and Sb in blood were below detection limits in 63.64%, 45.45%, 9.09%, 36.36%, 9.39%, 0.09% and 54.54% of the samples, respectively. Half the detection limits provided by CAF was used in the analysis (Hickey et al. 2001; Andreani et al. 2019). Barium was only detectable in three out of 11 fur samples with concentrations (μg g^−1^ w.w) of 1.07, 1.51 and 2.26, respectively. In all blood samples, Ba was below the detection limit and was therefore not included in the statistical analyses.

**Table 2:** Medians and ranges of the concentration of heavy metals and trace elements in the fur (μg g^−1^ w.w) and blood (μg ml^−1^ w.w) of molossid bats sampled on the Venetia Diamond Mine and Corea Game Farm (reference area). *n* = number of samples per tissue type and per site. The limit of quantification (LOQ) of elements in blood is indicated in brackets next to each relevant element symbol

	Fur (*n*=11)						Blood (*n*=11)					
Element (LOQ)	Corea Game Farm (*n*=5)			Venetia Diamond Mine (*n*=6)			Corea Game Farm (*n*=5)			Venetia Diamond Mine (*n*=6)		
	Median	Min	Max	Median	Min	Max	Median	Min	Max	Median	Min	Max
B (0.16)	3.380	1.643	3.914	7.440	6.791	18.900	1.330	1.250	2.321	2.162	1.250	3.015
Al (0.21)	102.620	51.437	558.062	109.380	43.969	185.393	0.11	0.105	1.999	0.11	0.105	1.380
K (12.7)	1330.06	769.00	2137.00	3310.00	2536.00	7298.00	2390.00	1882.00	3416.00	2450.00	1882.00	3379.00
Ca (0.2)	380.00	271.00	822.00	420.00	315.00	834.00	60.00	46.00	82.00	69.00	46.00	220.00
V (0.0004)	0.520	0.339	1.236	0.280	0.240	0.402	0.001	0.001	0.004	0.001	0.001	0.013
Cr (0.014)	1.070	0.369	2.708	0.990	0.545	1.471	0.060	0.014	0.099	0.007	0.007	0.409
Mn (0.011)	13.330	6.189	32.612	4.750	3.232	8.010	0.150	0.080	0.347	0.052	0.030	0.425
Fe (0.092)	147.64	79.05	814.00	101.41	67.54	181.25	629.47	484.83	861.64	690.91	631.09	1026.21
Co (0.0006)	0.240	0.166	0.673	0.130	0.096	0.199	0.001	0.0003	0.004	0.002	0.0003	0.004
Ni (0.004)	1.380	0.766	2.575	1.130	0.790	2.970	0.010	0.003	0.014	0.009	0.002	0.100
Cu (0.011)	6.090	5.343	17.413	8.430	5.686	26.106	0.380	0.261	0.665	0.731	0.261	0.968
Zn (0.0089)	97.700	37.775	241.865	147.090	117.801	346.409	1.900	1.376	3.589	4.040	1.376	7.044
As (0.0015)	0.260	0.184	0.320	0.720	0.369	1.201	0.005	0.003	0.009	0.006	0.003	0.023
Se (0.0009)	4.030	1.948	6.396	5.480	3.645	8.214	0.600	0.570	0.731	0.822	0.570	1.052
Rb (0.0006)	0.890	0.628	1.055	1.260	1.114	2.958	1.020	0.826	2.363	0.893	0.977	1.570
Sr (0.0009)	1.710	1.369	3.651	1.440	1.018	1.854	0.060	0.027	0.078	0.079	0.027	0.383
Mo (0.0012)	0.720	0.518	0.817	0.850	0.586	0.917	0.003	0.002	0.006	0.003	0.0006	0.007
Cd (0.0002)	0.020	0.016	0.097	0.090	0.031	0.171	0.0003	0.0001	0.003	0.001	0.0001	0.004
Sn (0.0012)	0.060	0.033	0.164	0.090	0.062	0.183	0.003	0.002	0.003	0.004	0.002	0.722
Sb (0.0006)	0.040	0.019	0.074	0.180	0.094	0.223	0.0003	0.0003	0.0003	0.001	0.0003	0.003
Hg (0.0005)	0.600	0.386	1.112	1.270	0.578	1.947	0.011	0.006	0.026	0.029	0.006	0.045
Pb (0.0017)	0.480	0.420	1.465	0.450	0.156	0.936	0.011	0.008	0.038	0.033	0.008	0.169

**Fig. 2:**
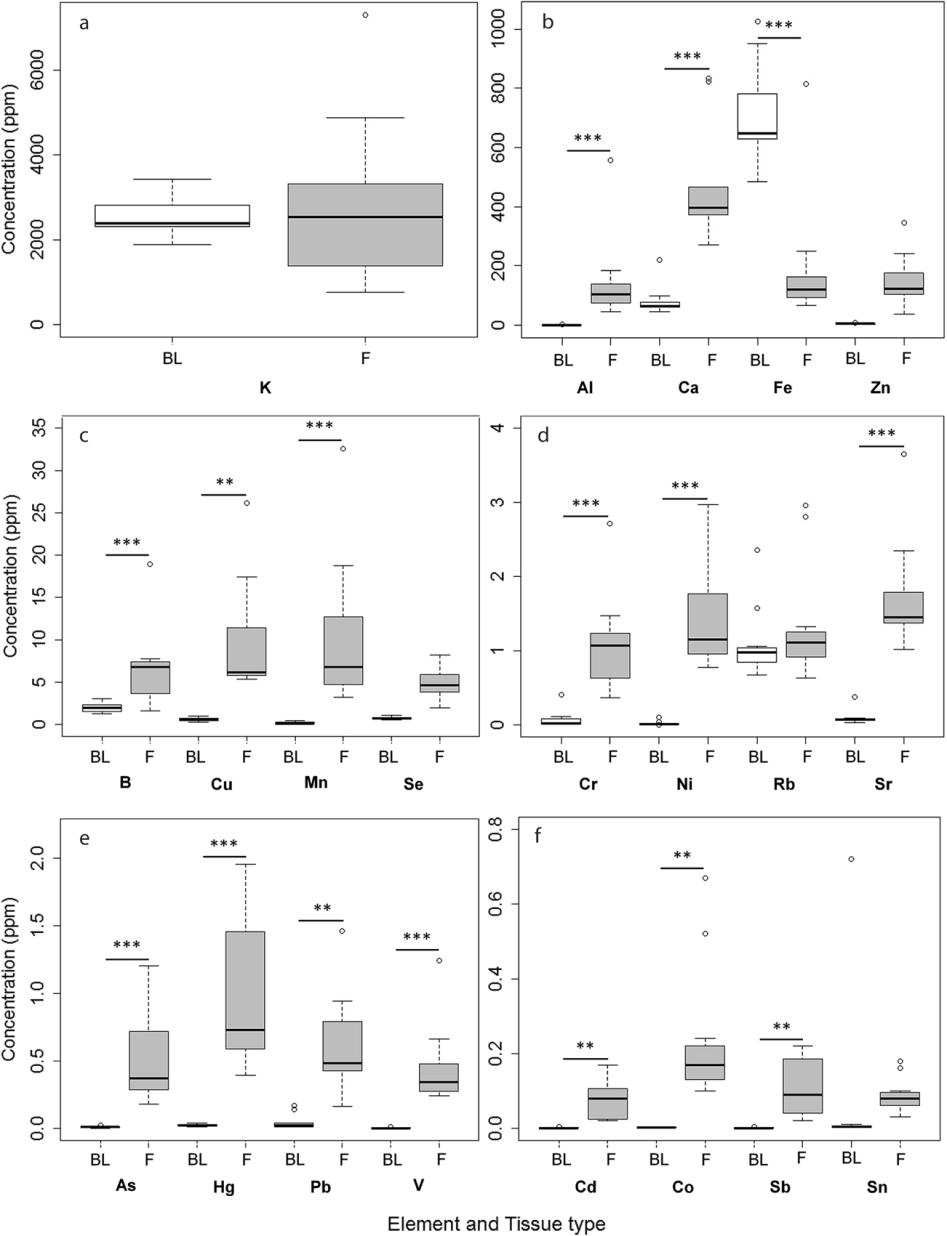
Box and whiskers plots indicating the median, the 25th and 75th percentiles and range of the concentrations of heavy metals and trace elements (open circles) in relation to tissue type (f=fur, b=blood). Statistically significant differences between heavy metal and trace element concentrations in fur and blood are indicated as follows: *= *P* < 0.05, **=*P* < 0.005, and ***P < 0.0005. Wilcoxon tests were used to compare Al, Ca, V, Cr, Mn, Ni, Rb and Sr; ANOVAs were used to compare K, Zn, Se; t-tests assuming unequal variances were used to compare B, Co, As, Cd, Sb, Hg and Pb and t-tests assuming equal variances were used to compare Fe, Cu and Sn

Concentrations of elements varied greatly for most elements (Table 2). Overall, fur and blood concentrations for most of the elements were fairly low except for fur concentrations of Al, Ca, Fe and Zn with overall median concentrations (μg g^−1^ w.w) of 104.88, 396.52, 120.24 and 121.68, respectively (Table 2). With the exception of Fe where a higher concentration was recorded in blood than fur (*P* < 0.05), concentrations of elements were generally higher in fur than blood (Fig. 2a–f), and significantly so for elements Al, Ca and Fe (Fig. 2b); B, Cu and Mn (Fig. 2c); Cr, Ni and Sr (Fig. 2d); As, Hg, Pb and V (Fig. 2e); and Cd, Co and Sb (Fig. 2f) (*P* < 0.05, Table 3).

**Table 3:** Summary of the statistical analyses *P*–values indicating significant (*P* < 0.05) and non-significant (*P* > 0.05) results in elements comparing element concentrations in fur and blood between sites and the total fur and blood concentrations. *CGF* control site; Corea Game Farm; *VDM* Venetia diamond mine

	Fur	Blood	Total
Element	CGF~VDM	CGF~VDM	Fur~Blood
B	*P* < 0.05^a^	*P* > 0.05^b^	*P* < 0.05^c2^
Al	*P* > 0.05^c1^	*P* > 0.05*^a^	*P* < 0.05^d^
K	*P* < 0.05^b^	*P* > 0.05^b^	*P* > 0.05^b^
Ca	*P* > 0.05^c1^	*P* > 0.05*^c1^	*P* < 0.05^d^
V	*P* > 0.05*^b^	*P* > 0.05^a^	*P* < 0.05^d^
Cr	*P* > 0.05^b^	*P* > 0.05*^c1^	*P* < 0.05^d^
Mn	*P* > 0.05^b^	*P* < 0.05*^c1^	*P* < 0.05^d^
Fe	*P* > 0.05^c1^	*P* > 0.05^b^	*P* < 0.05^c1^
Co	*P* > 0.05*^b^	*P* > 0.05*^c1^	*P* < 0.05*^c2^
Ni	*P* > 0.05^c1^	*P* > 0.05*^a^	*P* < 0.05^d^
Cu	*P* > 0.05^a^	*P* > 0.05^b^	*P* < 0.05^c1^
Zn	*P* > 0.05^c1^	*P* < 0.05^c1^	*P* > 0.05^b^
As	*P* > 0.05^b^	*P* > 0.05*^b^	*P* < 0.05^c2^
Se	*P* > 0.05^b^	*P* > 0.05*^b^	*P* > 0.05^b^
Rb	*P* < 0.05^c1^	*P* > 0.05^c1^	*P* > 0.05^d^
Sr	*P* > 0.05^b^	*P* > 0.05*^c1^	*P* < 0.05^d^
Mo	*P* > 0.05^c1^	*P* > 0.05^b^	*P* > 0.05*^b^
Cd	*P* < 0.05*^c1^	*P* > 0.05^c1^	*P* < 0.05^c2^
Sn	*P* > 0.05*^b^	*P* > 0.05*^a^	*P* > 0.05*^c1^
Sb	*P* > 0.05*^b^	*P* > 0.05*^b^	*P* < 0.05*^c2^
Hg	*P* > 0.05^c2^	*P* < 0.05*^c1^	*P* < 0.05^c2^
Pb	*P* > 0.05^c1^	*P* > 0.05^c1^	*P* < 0.05*^c2^

Since the data distributions were not all normally distributed; where both data sets were not normally distributed, Mann-Whitney*U*-test was performed (^a^) where both data sets were not normally distributed, ANOVAs were used (^b^) where both data sets were normally distributed, *t*-tests assuming equal (^c1^) and unequal variances (^c2^) were run where one data set was normally distributed and the other was not and a paired Wilcoxon test were run (^d^) when comparing the fur and blood concentrations of elements that were not normally distributed

*Cannot compute exact p-values with ties (data with the same values)

The bats from the mining footprint had significantly higher fur concentrations of B, Cd, K and Rb (*P* ≤ 0.05) than those from the reference area (Table 3). The maximum concentrations (μg g^−1^ w.w) were 18.9, 7.30, 2.96 and 0.171, respectively, from the mine. Although not statistically significant, it is interesting to note that bats fur from the reference area had higher maximum concentrations of Al (558.06 μg g^−1^), V (1.24 μg g^−1^), Cr (2.71 μg g^−1^), Mn (32.61 μg g^−1^), Fe (814.00 μg g^−1^), Co (0.673 μg g^−1^), Sr (3.651 μg g^−1^) and Pb (1.465 μg g^−1^) than the fur from bats roosting on the mine.

Blood element concentrations were comparable between the two sites except for Mn, Zn and Hg. The median concentration of Mn was significantly higher in the blood of the bats from the reference area (*P* < 0.05), even though the maximum concentration (μg ml^−1^ w.w) of 0.42 was recorded from an individual from the mine (Tables 2 and 3). Zinc and Hg were significantly higher (*P* < 0.05) in the blood of the bats from the Venetia Diamond Mine with maximum concentrations (μg ml^−1^ w.w) of 7.04 and 0.05 recorded from the blood of bats roosting on the mine (Tables 2 and 3). The highest concentration of a heavy metal was recorded for Fe in blood that ranged from a minimum concentration of 484.83 μg ml^−1^ from the reference area to 1026.21 μg ml^−1^ from the mine. Statistically, there was no difference in blood and fur Fe concentrations between the reference area and mining footprint.

The only significant correlations between fur and blood element concentration was for Hg; rho = 0.69, *P* < 0.05 and Sb; rho = 0.75, *P* < 0.05 (Table 4).

**Table 4:** Spearman’s Correlation analysis between total fur and blood element concentrations

Element	rho	*P*-value	S
B	0.40	0.23	132
Al	0.11	0.76	196.84
K	−0.45	0.17	318
Ca	0.23	0.50	170
V	−0.40	0.23	308
Cr	−0.06	0.86	233.31
Mn	0.13	0.71	192
Fe	−0.06	0.86	234
Co	−0.47	0.14	324.24
Ni	−0.08	0.82	238
Cu	0.09	0.80	200
Zn	0.55	0.09	100
As	0.50	0.12	110
Se	0.01	0.99	218
Rb	−0.29	0.39	284
Sr	−0.50	0.12	330
Mo	0.47	0.15	116
Cd	0.05	0.89	209.51
Sn	0.30	0.37	154
Sb	0.75	0.008	55.34
Hg	0.69	0.02	68
Pb	0.02	0.97	216

### Light trap and dietary analysis

**Fig. 3:**
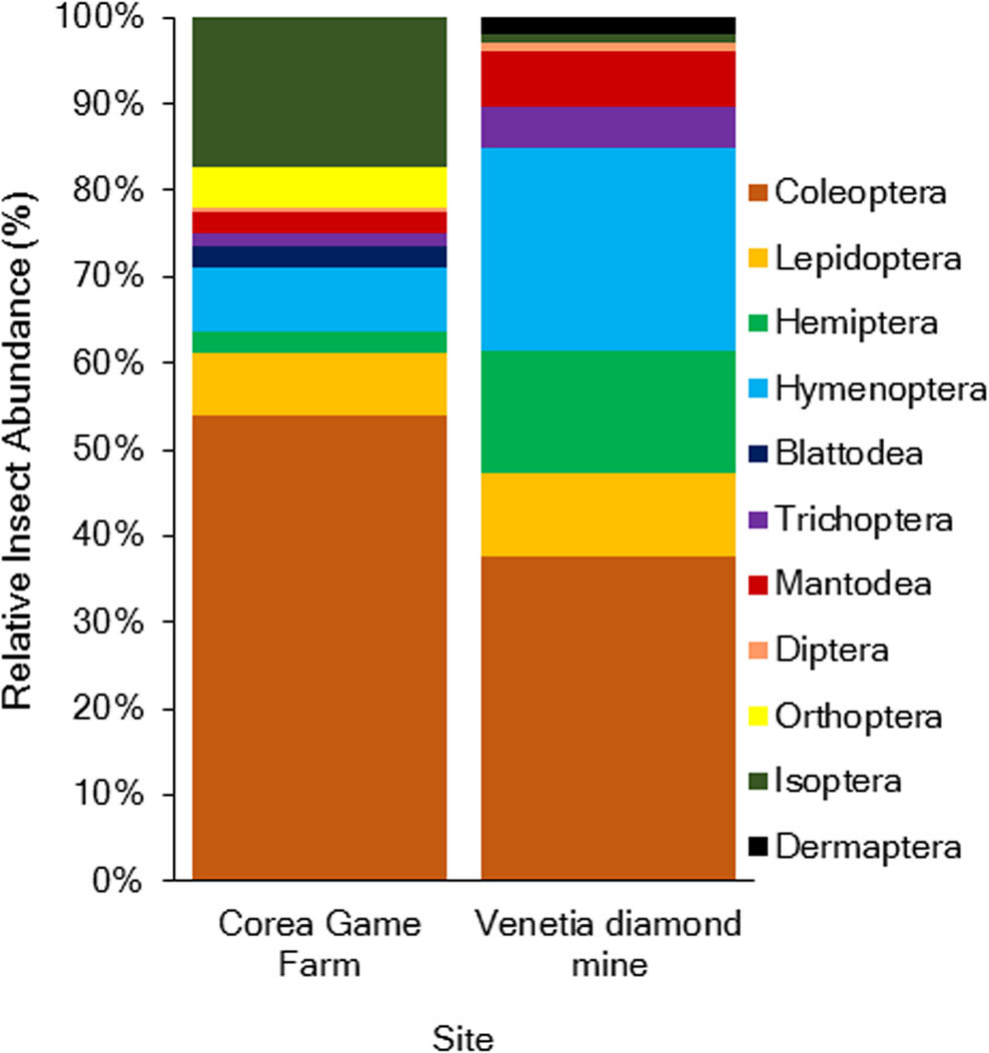
Relative abundance of insect orders sampled using a light trap on Corea Game Farm (*n* = 2 nights) and Venetia Diamond Mine (*n* = 2 nights) where Coleoptera were dominant on both sites comprising 53.92% and 37.74% of the sample, respectively

Insects collected from the light traps represented 11 orders; Coleoptera, Lepidoptera, Hemiptera, Hymenoptera, Blattodea, Trichoptera, Mantodea, Diptera, Orthoptera, Isoptera and Dermaptera (Fig. 3). The relative abundance of each order is represented in Fig. 2. Regarding light traps, Coleoptera dominated the light trap samples on both the mining and reference area comprising 53.92% and 37.74% of the sample, respectively (Fig. 3). Isoptera were the second dominant insect order only on the reference area comprising 17.16% of the sample, but their presence was negligible on the mine area (Fig. 3). Hymenoptera was the second most dominant order on the mining footprint (23.58%) followed by Hemiptera (14.15%) (Fig. 3). Isoptera were present in 20 of the 22 faecal pellets, while there is evidence of Coleoptera, Hymenoptera and Lepidoptera in one faecal pellet and of hemipteran in two faecal pellets (Supplementary information Table S1).

## Discussion

Although limited, we provide the first data for a range of 23 elements using fur and blood as biomarkers in two open-air forager bat species, *M. condylurus* and *T. aegyptiaca* from northern Limpopo Province, South Africa. Fifteen of the 22 elements investigated (excluding Ba) showed significant differences between fur and blood, with 14 elements being reported higher in fur than blood. Only Fe was found to be higher in blood than in the fur with approximately a seven fold difference in the medians between the two sites. Correlations between tissues and internal organs are not standard and cannot be generalised. In our study, a novel finding was that Sb and Hg had significant (although weak) correlations between fur and blood concentrations. The correlation between fur and blood Hg concentrations is supported by similar findings by Karouna-Renier et al. (2014) and Yates et al. (2014). They found that Hg concentrations in the blood of insectivorous bat species were highly predictive by fur Hg concentrations (Karouna-Renier et al. 2014; Yates et al. 2014). There are currently no toxic thresholds of heavy metals and trace elements for fur concentrations and internal organs/tissues in bats. In the available literature, reported element concentrations range from means to geometric means and medians, and thus, without the raw data, it is not always easily comparable across studies (for example; Hickey et al. 2001; Becker et al. 2018; Mina et al. 2019 and the current study). Comparative data for the 23 elements investigated in our study is scant and limited to a handful of studies that will be discussed below to place our findings into a global context.

The median levels of Hg in bat fur reported in our study for molossid bats are well below the concentrations responsible for neurological alterations in *M. lucifugus* (Becker et al. 2018)*.* Mercury concentrations in the molossid fur in our study are generally lower than those reported in insectivorous bats by Hickey et al. (2001), Karouna-Renier et al. 2014 and Becker et al. (2018), higher than those reported in skin-fur by Andreani et al. (2019) and are comparable with the range of Hg reported by other studies as a consequence of a variety of anthropogenic activities (Ferrante et al. 2018; Carrasco-Rueda et al. 2020). Mercury concentration in fur was shown to be lowest for frugivorous and nectivorous bat species from the family Phyllostomidae, followed in increasing concentrations of Hg in fur by omnivores, gleaning insectivores and carnivores (Carrasco-Rueda et al. 2020). Aerial insectivores (Vespertilionidae, Molossidae and Emballonuridae) had the highest concentration of Hg in their fur (Carrasco-Rueda et al. 2020). The authors suggested that the Hg levels may not pose a health risk and could be due to background concentrations in the environment and not as a result of proximity to gold mining activities or agricultural practices (Carrasco-Rueda et al. 2020). In Canada (Ontario and Quebec), Hickey et al. (2001) reported high and significantly different concentrations of Hg in the fur of *Myotis lucifugus*, *M. septentrionalis*, *M. leibii* and *Eptesicus fuscus* with values that could reflect biomagnification from contaminated aquatic prey. Wieringa et al. (2020) reported a lower mean Hg concentration in fur of *Lasiurus borealis* from across its range in North America than our study, which was similar to the concentrations reported for Phyllostomidae bats in Carrasco-Rueda et al. (2020). The concentrations of Hg in bat fur could indicate the level of environmental exposure and potential toxicological effects in individuals. Mercury has been reported to have a toxic threshold of ≥10 ppm (or 10 μg g^−1^) in hair that indicates adverse health effects such as neurochemical effects and functional behaviour changes in wild mice and captive mink (Wobeser et al. 1976; Burton et al. 1977) and has been accepted to have potential health effects in bats (Becker et al. 2018; Moreno-Brush et al. 2018; Carrasco-Rueda et al. 2020). Mercury contamination in the fur of comparatively few bat species from areas impacted by anthropogenic activities has been investigated. For example, 10 μg g^−1^ total Hg in the fur of *M. lucifugus* indicates neurological alterations but lower concentrations of Hg correlate to innate immunity in *Desmodus rotundus* (Becker et al. 2018). The low concentrations in the molossid fur in our study could indicate low exposure to Hg in their environment with low health risks.

Lead in the blood of one individual molossid roosting on the mine had a blood concentration which was hazardously close to the level of 0.2 ppm Pb contamination in the blood of an animal (cattle) considered to be lead poisoned (Reis et al. 2010). Additionally, if an animal is suffering from Pb poisoning, the animal should also be deficient in Ca, Fe and Zn; however, the individual bat of concern had the highest blood concentrations of Fe, Ca and Zn compared to all the other individuals. The implications of this observation are unknown. Elevated levels of Pb in the fur of bats could be due to Pb contamination of prey and the physical exposure of bats to vehicular traffic continually entering and exiting the mine throughout the day in close proximity to their roost as seen in *Pipistrellus pipistrellus* (Flache et al. 2015). In our study, the median Pb concentrations in the fur of the bats from both sites were similar and were comparable with median fur concentrations of *M. myotis* in Flache et al. (2015) and the range reported in Ferrante et al. (2018) in mixed environments. Our study presents Pb concentrations much lower than skin-fur concentrations reported by Andreani et al. (2019) in *T. teniotis* and *Miniopterus schreibersii* from Italy. Similarly, the maximum concentrations of Pb reported in our study are considerably lower than that reported for *Afronycteris nana* (previously *Neoromicia nana*) foraging over wastewater treatment works and from a reference site in South Africa (Hill et al. 2017).

The median and minimum fur Al level were slightly elevated in the reference area but maximum concentration recorded from the reference area was much higher than in the fur from the mine. Aluminium concentrations recorded in the fur of the molossids in our study were ~1.6 up to 23 times higher than the concentrations of Al in fur of *M. lucifugus* and *Eptesicus fuscus* that comparatively were very low (Hickey et al. 2001). Some of the Al values for *M. lucifugus* fell within the range of values from the mine. Andreani et al. (2019) reported a mean fur Al concentration in skin-fur samples of *T. teniotis* and *M. schreibersii* from a polluted area in Italy (African Quarter of Rome) that was higher than the median recorded in our study from the reference area, but lower than the maximum concentration reported at the same site. Aluminium is a non-essential element (Reis et al. 2010) and the range of concentrations in blood and fur of the molossids raises a cause of concern. In rodents, bone concentration of Al >10 μg g^−1^ has been considered to indicate a reduction in the ability of the animal to excrete Al or an indication of exposure to high concentrations of Al (Scheuhammer 1987). Aluminium is a common element in soils (Rosseland et al. 1990) and has been shown to be present in soil dust (8.2% according to Friedlander 1973) but the percentage that Al contributes to soil dust on the Venetia Diamond Mine and surrounding area is unknown. Future research should investigate whether these concentrations measured in the molossid fur indicate (1) the background levels, (2) a contamination event (either through dust or roosting structure) and (3) any health implications.

Levels of Fe reported in the fur for the reference area and mine bats were comparable with the concentration observed in *E. fuscus* in Hickey et al. (2001) but higher than the ranges reported for *A. nana* foraging over wastewater treatment works and from reference sites (Hill et al. 2017). The maximum concentration recorded from an individual from the reference area was the highest level reported in fur compared to available literature. The high Fe fur concentration could indicate external contamination, potentially from a roost in an anthropogenic structure, although the same individual had the third highest blood concentration of Fe. Median fur Zn concentrations were similar to the medians and ranges for *P. pipistrellus* (Flache et al. 2015) but higher than *M. nattereri*, *M. bechsteinii*, *M. daubentonii* and *Plectus auritus* studied by Flache et al. (2015, 2018), and comparable with *M. lucifugus*, *M. septentrionalis* and *E. fuscus* studied by Hickey et al. (2001). The ranges of Zn concentrations in our study also overlapped with concentrations reported in *A. nana* foraging over waste water and reference areas in Hill et al. (2017) although our minimum and maximum ranges were lower. Flache et al. (2015) had noted that *M. daubentonii* is known to forage on chironomid midges emerging from water bodies with contaminated sediment. Perhaps a similar occurrence took place with the molossids opportunistically feeding on emerging adult insects from the mine waste water dam that may explain the observed high Zn concentrations in the fur and blood of the bats from the mine. However, the high Zn concentration in fur that was reflected in the blood of the bats from the mine could have also been ingested through grooming activities if there was external contamination of the fur from dust or the roosting site.

The median As fur concentrations from the mine footprint and reference area were comparable to *T. teniotis* and *M. schreibersii* skin-fur from a polluted urban area in Italy (Andreani et al. 2019), *M. myotis* roosting near a petrochemical plant as well as in an uncontaminated (Ferrante et al. 2018), *Hypsugo savii/Nyctalus leisleri/P. pipistrellus/P. pygmaeus* from a wind farm in Portugal (Mina et al. 2019) and *A. nana* (Hill et al. 2017). Arsenic is an element of concern as it can crosses the blood-brain barrier and is implicated in neurogenerative diseases (Escudero-Lourdes 2016). Elevated concentrations of As in bat fur may indicate negative neurological effects in the long-term (Hill et al. 2017). Low and significantly different concentrations of fur Cd were reported for the molossids on the mine and in the reference area. These concentrations of Cd are higher than Cd medians in *M. myotis* (Ferrante et al. 2018) but comparable with medians for *H. savii/N. leisleri/P. pipistrellus/P. pygmaeus* (Mina et al. 2019), mean concentrations in fur of *A. nana* foraging over two wastewater treatment works and a reference area (Hill et al. 2017) and in skin-fur of *T. teniotis* and *M. schreibersii* (Andreani et al. 2019). Overall, the Cd values reported in our study overlap with the ranges reported in Flache et al. (2015), Hernout et al. (2016a) and Flache et al. (2018) from a variety of land uses excluding mines. The concentration of Sb in the bat fur from the mine was slightly elevated above the reference area and the opposite was observed for V. Vanadium was higher and Sb was slightly elevated above the median concentrations recorded for *M. myotis* in Sicily from a polluted area near a petrochemical plant and a control area in Pantalica (Ferrante et al. 2018) but were lower than the mean concentration of Sb recorded in skin-fur of *T. teniotis* and *M. schreibersii* (Andreani et al. 2019).

The range of fur concentrations of Co, Ni, Se and Mn of the molossids from both sites in our study was most similar to levels reported in *H. savii/N. leisleri/P. pipistrellus/P. pygmaeus* (Mina et al. 2019), but our maximum values were higher than reported by Ferrante et al. (2018). Manganese concentrations in the fur of the molossids also fell within the ranges reported for *A. nana* foraging over wastewater treatment works but the maximum concentration reported from the mine in our study was nearly double the maximum concentration recorded from the reference sites in Hill et al. (2017). Median Cu concentrations in the molossids fur at both sites were up to 6.5 times higher than the values in *M. myotis* (Ferrante et al. 2018). The ranges of the molossid fur Cu concentrations fell within the ranges reported by Flache et al. (2015), Hernout et al. (2016b), Hill et al. (2017), Flache et al. (2018) and Mina et al. (2019). The median fur concentration of Mo in *M. bechsteinii* (Flache et al. 2018) was lower to that determined in the molossids from the mine and reference area with some overlap in ranges. The only comparative data for fur Sn and Rb concentrations in the literature have recently been published by Wieringa et al. (2020). Mean fur concentrations of Sn and Rb (Wieringa et al. 2020) were lower than the minimum values recorded from the mine and reference area. Comparative concentrations of Sr are presented in Andreani et al. (2019) with a skin-fur mean concentration 2.5 and 5 times higher than the maximum concentrations reported in our study in the molossid bats fur from the reference area and mine footprint. The concentration range of Ba in the fur of the molossids in our study was higher than reported in lactant *T. teniotis* across its range but lower than *T. teniotis/M. schreibersii* skin-fur samples (Andreani et al. 2019).

Lastly, Cr levels in the molossids in our study from the reference area and from the mine footprint were comparable to the range of concentrations in *H. savii/N. leisleri/P. pipistrellus/P. pygmaeus* on wind farms in Portugal (Mina et al. 2019), and in *M. myotis* from a control area and an area near a petrochemical plant, Sicily (Ferrante et al. 2018). Similarly, the molossid fur concentrations of Cr showed some overlap with the ranges reported for *A. nana* captured over wastewater treatment works and reference sites in South Africa (Hill et al. 2017). Currently, there are no comparable studies for the bat fur concentrations of B and K with other free-ranging insectivorous bat species; thus, the interpretation and potential health impacts of these element concentrations remains unknown.

Data are scant concerning element levels in free-ranging small mammal blood for an adequate comparison with the current data. The mean Hg concentration in the blood from the molossids from the mine in our study was comparable with the concentration reported in *M. lucifugus* from Moscow by Karouna-Renier et al. (2014). Median blood Hg concentration from the molossids collected from our reference area was lower than the reference and contaminated sites reported by Karouna-Renier et al. (2014), notably lower than reported in Yates et al. (2014) and comparable with whole bat samples in Andreani et al. (2019). Significantly higher levels of mtDNA damage were reported in the bats from the contaminated areas in Virginia, USA (Karouna-Renier et al. 2014). The low concentrations of Hg in our study potentially indicate negligible health risks. A study on whole bat samples (Smith and Rongstad 1982) investigating Zn, Cu, Cd, Pb and Ni concentrations in bats from a proposed mining site and an active mine near Timmins, Ontario, Canada, revealed heavy metal concentrations much higher than those recorded in the blood from the molossids in our study. Similarly, Andreani et al. (2019) reported Al, As, Ba, Cd, Pb, Sb and Sr concentrations in whole samples that were higher than those in our molossid blood. Certain elements are known to accumulate in the internal organs and tissues (Naidoo et al. 2016); thus, the whole bat sample would have much higher concentrations than the blood samples, but we use it as a proxy nonetheless. With the exception of Fe and Zn, the reported element values in the blood of the molossids may reflect the background conditions; future research in this area is critical to establish baseline reference data in South Africa.

*Tadarida aegyptiaca* predominantly feeds on Coleoptera (including water beetles), Lepidoptera, Orthoptera, Hymenoptera, Isoptera, Diptera and Arachnids (spiders) (Taylor et al. 2019; Monadjem et al. 2020). *Mops condylurus* feeds mainly on Coleoptera, Hemiptera, Diptera and Lepidoptera (Taylor et al. 2019; Monadjem et al. 2020) and during sample collection, fed mostly on Isoptera that emerged in response to the onset of the rainfall season. This suggests that the bats roosting on the mine were not foraging in the vicinity of the mist net capture sites on the mine where the insect sampling took place but possibly over the natural areas adjacent to the mine. It is known that elements bioaccumulate through trophic levels (Pikula et al. 2010; Ali et al. 2019) but concentrations in bat diets are largely unknown. In general, the diet of different bat species has been shown in the literature to be an important route through which elements could accumulate in tissues and organs of bats (Karouna-Renier et al. 2014; Becker et al. 2018; Carrasco-Rueda et al. 2020). Idowu et al. (2014) and Denloye et al. (2015) presented low concentrations of heavy metal accumulation in mound termites (Termitidae) that are fondly consumed by people in Africa. The latter authors cautioned that even though the concentrations elements in the different species and castes were low, the different species and castes of termite may have different abilities to accumulate heavy metals based on their physiological needs and could pose a risk to humans regularly consuming them. Termites may provide a seasonal source of element ingestion in molossid bats and other bat species that opportunistically feed on them during the summer emergence.

The only available literature concerning elemental concentrations in South African bats was published on *A. nana*, by Naidoo et al. (2013, 2015, 2016) and Hill et al. (2016, 2017) focusing on health impacts of bioaccumulation. Bioaccumulation of heavy metals in *A. nana* could have been through the consumption of Diptera (possibly chironomid midges that are tolerant of the polluted water bodies) swarming over the waste water sites (Naidoo et al*.* 2013). The addition of element concentrations in fur and blood of molossid bats provide some important data to further investigate the use of non-invasive methods to use bats as bioindicators of element exposure whether it be through exposure through drinking water, ingesting contaminated prey items or exposure in roosts.

### Implications for bat conservation

In addition to the global stressors that bats already face, namely habitat loss, changes in available resource quantity and quality, climate change, increasing number of wind turbines, disease pressure (Hernout et al. 2016b; Flache et al. 2018; de Souza et al. 2020; Lawson et al. 2020) and environmental pollution including organophosphates (Bayat et al. 2014), it is vital that both the origin of elements and the toxicological response of bats is understood. This knowledge will assist conservation authorities and specialists to make informed decisions concerning bat conservation and mitigation strategies within the context of looming anthropogenic developments and conservation areas. For example, we may find that in accordance with legislation and mine protocols, the current specifications for the containment of waste water can still expose ecosystems to elevated concentrations of elements. Bats could be used bioindicators of environmental change by using fur as a biomarker that could indicate bioaccumulation of elements through bat activity/behaviour (drinking, foraging, grooming) over areas impacted by anthropogenic activities. The shortfall with the current available literature is that we still do not know (1) the specific toxicological thresholds for numerous bat species, (2) the physiological and resulting ecological effects of these elements and (3) whether there are phylogenetic responses to elements (e.g. total mercury concentrations in fur has been shown to have a strong phylogenetic signal as reported by Becker et al. (2018)). Southern hemisphere bat species have been poorly studied in this regard. Additionally, there is a lack of long-term and recapture studies on the impact of heavy metals and trace elements on bat populations that include the effects of age and sex (Hernout et al. 2016a).

## Conclusion

Only six out of the 23 elements tested in the fur and blood were significantly higher in the bats roosting on the mine compared to those from the reference area namely; B, K, Rb and Cd (fur) and Zn and Hg (blood). Manganese (blood) was significantly higher in the bats from the reference area than those roosting on the mine. Overall, 16 elements significantly differed between fur and blood, with most elements except Fe present in higher concentrations in fur than blood. Only Sb and Hg concentrations had significant correlations between fur and blood providing support that for at least these two elements, fur could provide a reliable indication of internal element concentrations.

The concentrations of the elements reported in our study could reflect the natural background levels. However, this may fluctuate during the year, which remains to be tested. The impacts of these elements on the organs and bones of bats also remain to be tested and may reveal another trend. We could not investigate element contamination of all bat species in the study area, as comparative individuals were not captured; thus, we do not know the impact of the mining activity on other bat species known to occur in the area. Future investigation is required in this regard. There are additional potential sources of contamination that need to be investigated, such as potential exposure to elements based on roost selection, for example roosting in metal infrastructure on the mine and surrounding farms versus roosting in a natural roost (rock crevice) and possible ingestion of elements through grooming. The data presented here should be used with caution and can be regarded as the first baseline data for two species of molossid bats in northern Limpopo, South Africa.

## Supplementary information

ESM 1 (DOCX 62 kb)

## Data Availability

The datasets used and/or analysed during our study are available from the corresponding author on reasonable request.
