# Supplementary material for: Non-invasive sampling of bats reflects their potential as ecological indicators of elemental exposure in a diamond mining area, northern Limpopo Province, South Africa
Source: Environ Sci Pollut Res Int. 2021 Sep 30;29(9):13647–60. doi: 10.1007/s11356-021-16466-x (PMC8803726; doi:10.1007/s11356-021-16466-x)
Supplement: Supplementary file 1 — (DOCX 62 kb) [file 11356_2021_16466_MOESM1_ESM.docx]

| **Table S1.** Selected individuals of *Mops condylurus* and *Tadarida aegyptiaca* captured during summer (December 2018) from the Venetia diamond mine (VDM) and Corea Game Farm (CGF) with their associated measurements and faecal pellet analysis data. Non-reproductive females indicated by “NR” | | | | | | | | | |
| --- | --- | --- | --- | --- | --- | --- | --- | --- | --- |
|  |  |  |  |  | Percentage frequency of insect order (Family/Suborder) | | | | |
| Location (Bat Code) | Sex | Forearm Length (mm) | Mass (g) | Species (# of faecal pellets) | Isoptera  (Termitidae) | Hymenoptera  (Formicidae) | Coleoptera | Lepidoptera | Hemiptera  (Heteroptera) |
| VDM (DCT024) | Male | 47.11 | 21.50 | *M. condylurus* (n=1) | 100 | - | - | - | - |
| VDM (DCT025) | Male | 47.13 | 23.00 | *M. condylurus* (n=2) | 100 | - | - | - | - |
| VDM (DCT029) | Female (NR) | 43.99 | 18.00 | *M. condylurus* (n=2) | 100 | - | - | - | - |
| VDM (DCT033) | Male | 46.90 | 23.00 | *M. condylurus* (n=2) | 100 | - | - | - | - |
| VDM (DCT034) | Female (NR) | 46.22 | 20.00 | *M. condylurus* (n=1) | 100 | - | - | - | - |
| VDM (DCT035) | Male | 49.82 | 26.00 | *M. condylurus* (n=3) | 100 | - | - | - | - |
| CGF (DCT049) | Female (NR) | 46.58 | 18.00 | *T. aegyptiaca* (n=4) | 99 | 1 | - | - | - |
| CGF (DCT051) | Female (NR) | 47.91 | 19.50 | *T. aegyptiaca* (n=4) | 100 | - | - | - | - |
| CGF (DCT052) | Female (NR) | 48.54 | 18.50 | *T. aegyptiaca* (n=1) | 100 | - | - | - | - |
| CGF (DCT066) | Female (NR) | 45.83 | 22.00 | *M. condylurus* (n=1) | - | - | - | 90 | 10 |
| CGF (DCT070) | Female (NR) | 48.06 | 24.50 | *T. aegyptiaca* (n=1) | - | - | 99.5 | - | 0.5 |

| **Table S2.** Fur and blood concentrations (μg.g^-1^ wet-weight) from the current study and literature for comparative purposes. References marked with “*” indicate dry weight values converted to wet weight for comparative purposes with the current data | | | | | | | | | | |
| --- | --- | --- | --- | --- | --- | --- | --- | --- | --- | --- |
| Species (Insectivorous only) | Region | Sector/Feature | Biological Material | Element | Mean W. W | Median W. W | Geometric Mean | Min W.W | Max W.W | Reference |
| *Mops condylurus* | Northern Limpopo, SA | Diamond Mining | Blood | Al Semi Quantitative |  | 0.11 |  | 0.11 | 1.38 | Current study |
| *Tadarida aegyptiaca/Mops condylurus* | Northern Limpopo, SA | Game Farm | Blood | Al Semi Quantitative |  | 0.11 |  | 0.11 | 2.00 | Current study |
| *Mops condylurus* | Northern Limpopo, SA | Diamond Mining | Blood | As |  | 0.006 |  | 0.003 | 0.02 | Current study |
| *Tadarida aegyptiaca/Mops condylurus* | Northern Limpopo, SA | Game Farm | Blood | As |  | 0.005 |  | 0.003 | 0.01 | Current study |
| *Mops condylurus* | Northern Limpopo, SA | Diamond Mining | Blood | B |  | 2.16 |  | 1.25 | 3.01 | Current study |
| *Tadarida aegyptiaca/Mops condylurus* | Northern Limpopo, SA | Game Farm | Blood | B |  | 1.33 |  | 1.25 | 2.32 | Current study |
| *Mops condylurus* | Northern Limpopo, SA | Diamond Mining | Blood | Ba |  | 0.03 |  | - | - | Current study |
| *Tadarida aegyptiaca/Mops condylurus* | Northern Limpopo, SA | Game Farm | Blood | Ba |  | 0.03 |  | - | - | Current study |
| *Mops condylurus* | Northern Limpopo, SA | Diamond Mining | Blood | Ca |  | 0.07 |  | 0.05 | 0.22 | Current study |
| *Tadarida aegyptiaca/Mops condylurus* | Northern Limpopo, SA | Game Farm | Blood | Ca |  | 0.06 |  | 0.05 | 0.08 | Current study |
| *Mops condylurus* | Northern Limpopo, SA | Diamond Mining | Blood | Cd |  | 0.001 |  | 0.0001 | 0.004 | Current study |
| *Tadarida aegyptiaca/Mops condylurus* | Northern Limpopo, SA | Game Farm | Blood | Cd |  | 0.0003 |  | 0.0001 | 0.003 | Current study |
| *Mops condylurus* | Northern Limpopo, SA | Diamond Mining | Blood | Co |  | 0.002 |  | 0.0003 | 0.004 | Current study |
| *Tadarida aegyptiaca/Mops condylurus* | Northern Limpopo, SA | Game Farm | Blood | Co |  | 0.001 |  | 0.0003 | 0.004 | Current study |
| *Mops condylurus* | Northern Limpopo, SA | Diamond Mining | Blood | Cr |  | 0.007 |  | 0.01 | 0.41 | Current study |
| *Tadarida aegyptiaca/Mops condylurus* | Northern Limpopo, SA | Game Farm | Blood | Cr |  | 0.060 |  | 0.01 | 0.10 | Current study |
| *Mops condylurus* | Northern Limpopo, SA | Diamond Mining | Blood | Cu |  | 0.73 |  | 0.26 | 0.97 | Current study |
| *Tadarida aegyptiaca/Mops condylurus* | Northern Limpopo, SA | Game Farm | Blood | Cu |  | 0.38 |  | 0.26 | 0.66 | Current study |
| *Mops condylurus* | Northern Limpopo, SA | Diamond Mining | Blood | Fe |  | 690.91 |  | 631.09 | 1026.21 | Current study |
| *Tadarida aegyptiaca/Mops condylurus* | Northern Limpopo, SA | Game Farm | Blood | Fe |  | 629.47 |  | 484.83 | 861.64 | Current study |
| *Mops condylurus* | Northern Limpopo, SA | Diamond Mining | Blood | Hg |  | 0.03 |  | 0.01 | 0.04 | Current study |
| *Tadarida aegyptiaca/Mops condylurus* | Northern Limpopo, SA | Game Farm | Blood | Hg |  | 0.01 |  | 0.01 | 0.03 | Current study |
| *Perimyotis subflavus* | Northeast United States | Point source and non-point source | Blood | Hg | 40.77 | 15.3 |  |  | 255.00 | Yates et al. 2014 |
| *Myotis lucifugus* | Northeast United States | Point source and non-point source | Blood | Hg | 29.22 | 5.39 |  |  | 707.64 | Yates et al. 2014 |
| *Myotis septentionalis* | Northeast United States | Point source and non-point source | Blood | Hg | 26.89 | 7.37 |  |  | 480.00 | Yates et al. 2014 |
| *Myotis grisescens* | Northeast United States | Point source and non-point source | Blood | Hg | 18.61 | 5.37 |  |  | 84.50 | Yates et al. 2014 |
| *Eptesicus fuscus* | Northeast United States | Point source and non-point source | Blood | Hg | 16.64 | 9.59 |  |  | 200.00 | Yates et al. 2014 |
| *Myotis sodalis* | Northeast United States | Point source and non-point source | Blood | Hg | 10.58 | 10.35 |  |  | 18.30 | Yates et al. 2014 |
| *Myotis leibii* | Northeast United States | Point source and non-point source | Blood | Hg | 12.88 | 15.70 |  |  | 18.83 | Yates et al. 2014 |
| *Lasionycteris noctivagans* | Northeast United States | Point source and non-point source | Blood | Hg | 7.96 | 7.89 |  |  | 14.23 | Yates et al. 2014 |
| *Lasiurus borealis* | Northeast United States | Point source and non-point source | Blood | Hg | 4.03 | 2.73 |  |  | 25.54 | Yates et al. 2014 |
| *Lasiurus cinereus* | Northeast United States | Point source and non-point source | Blood | Hg | 1.33 | 1.34 |  |  | 3.61 | Yates et al. 2014 |
| *Myotis lucifugus* | Moscow, Russia | Uncontaminated | Blood | Hg | 0.03 |  |  | 0.005 | 0.61 | Karouna-Renier et al. 2014 |
| *Myotis lucifugus* | Northwestern Virginia, USA | Contaminated (Grottoes & Mt. Sydney | Blood | Hg | 0.74 |  |  | 0.014 | 3.80 | Karouna-Renier et al. 2014 |
| *Myotis lucifugus* | Northwestern Virginia, USA | Contaminated (Grottoes) | Blood | Hg | 1.39 |  |  | 0.08 | 3.76 | Karouna-Renier et al. 2014 |
| *Myotis lucifugus* | Northwestern Virginia, USA | Contaminated (Mt. Sydney | Blood | Hg | 0.11 |  |  | 0.014 | 0.92 | Karouna-Renier et al. 2014 |
| *Mops condylurus* | Northern Limpopo, SA | Diamond Mining | Blood | K |  | 2.45 |  | 1.88 | 3.38 | Current study |
| *Tadarida aegyptiaca/Mops condylurus* | Northern Limpopo, SA | Game Farm | Blood | K |  | 2.39 |  | 1.88 | 3.42 | Current study |
| *Mops condylurus* | Northern Limpopo, SA | Diamond Mining | Blood | Mn |  | 0.05 |  | 0.08 | 0.42 | Current study |
| *Tadarida aegyptiaca/Mops condylurus* | Northern Limpopo, SA | Game Farm | Blood | Mn |  | 0.15 |  | 0.08 | 0.35 | Current study |
| *Mops condylurus* | Northern Limpopo, SA | Diamond Mining | Blood | Mo |  | 0.003 |  | 0.001 | 0.01 | Current study |
| *Tadarida aegyptiaca/Mops condylurus* | Northern Limpopo, SA | Game Farm | Blood | Mo |  | 0.003 |  | 0.002 | 0.01 | Current study |
| *Mops condylurus* | Northern Limpopo, SA | Diamond Mining | Blood | Ni |  | 0.009 |  | 0.002 | 0.10 | Current study |
| *Tadarida aegyptiaca/Mops condylurus* | Northern Limpopo, SA | Game Farm | Blood | Ni |  | 0.01 |  | 0.003 | 0.01 | Current study |
| *Mops condylurus* | Northern Limpopo, SA | Diamond Mining | Blood | Pb |  | 0.033 |  | 0.01 | 0.17 | Current study |
| *Tadarida aegyptiaca/Mops condylurus* | Northern Limpopo, SA | Game Farm | Blood | Pb |  | 0.011 |  | 0.01 | 0.04 | Current study |
| *Mops condylurus* | Northern Limpopo, SA | Diamond Mining | Blood | Rb |  | 0.893 |  | 0.98 | 1.57 | Current study |
| *Tadarida aegyptiaca/Mops condylurus* | Northern Limpopo, SA | Game Farm | Blood | Rb |  | 1.020 |  | 0.83 | 2.36 | Current study |
| *Mops condylurus* | Northern Limpopo, SA | Diamond Mining | Blood | Sb |  | 0.001 |  | 0.0003 | 0.003 | Current study |
| *Tadarida aegyptiaca/Mops condylurus* | Northern Limpopo, SA | Game Farm | Blood | Sb |  | 0.000 |  | 0.0003 | 0.0003 | Current study |
| *Mops condylurus* | Northern Limpopo, SA | Diamond Mining | Blood | Se |  | 0.822 |  | 0.57 | 1.05 | Current study |
| *Tadarida aegyptiaca/Mops condylurus* | Northern Limpopo, SA | Game Farm | Blood | Se |  | 0.600 |  | 0.57 | 0.73 | Current study |
| *Mops condylurus* | Northern Limpopo, SA | Diamond Mining | Blood | Sn |  | 0.004 |  | 0.002 | 0.72 | Current study |
| *Tadarida aegyptiaca/Mops condylurus* | Northern Limpopo, SA | Game Farm | Blood | Sn |  | 0.003 |  | 0.002 | 0.003 | Current study |
| *Mops condylurus* | Northern Limpopo, SA | Diamond Mining | Blood | Sr |  | 0.079 |  | 0.03 | 0.38 | Current study |
| *Tadarida aegyptiaca/Mops condylurus* | Northern Limpopo, SA | Game Farm | Blood | Sr |  | 0.060 |  | 0.03 | 0.08 | Current study |
| *Mops condylurus* | Northern Limpopo, SA | Diamond Mining | Blood | V |  | 0.001 |  | 0.001 | 0.01 | Current study |
| *Tadarida aegyptiaca/Mops condylurus* | Northern Limpopo, SA | Game Farm | Blood | V |  | 0.001 |  | 0.001 | 0.004 | Current study |
| *Mops condylurus* | Northern Limpopo, SA | Diamond Mining | Blood | Zn |  | 4.04 |  | 1.38 | 7.04 | Current study |
| *Tadarida aegyptiaca/Mops condylurus* | Northern Limpopo, SA | Game Farm | Blood | Zn |  | 1.90 |  | 1.38 | 3.59 | Current study |
| *Myotis lucifugus* | Lafleche-1 Cave, Quebec, Canada | Cave | Fur | Al | 67.00 |  |  |  |  | Hickey et al. 2001 |
| *Myotis lucifugus* | Lafleche 2 Cave, Quebec, Canada | Cave | Fur | Al | 70.00 |  |  |  |  | Hickey et al. 2001 |
| *Myotis lucifugus* | Tyendinaga, Ontario, Canada | Mixed urban | Fur | Al | 41.00 |  |  |  |  | Hickey et al. 2001 |
| *Myotis lucifugus* | Fly Creek, Ontario, Canada | Mixed urban | Fur | Al | 27.00 |  |  |  |  | Hickey et al. 2001 |
| *Eptesicus fuscus* | Cornwall, Ontario, Canada | Mixed urban | Fur | Al | 4.80 |  |  |  |  | Hickey et al. 2001 |
| *Lasiurus borealis* | North America | Across range | Fur | Al | 0.02 |  |  |  |  | Wieringa et al. 2020 |
| *Mops condylurus* | Northern Limpopo, South Africa | Diamond Mining | Fur | Al Semi Quantitative |  | 109.38 |  | 43.97 | 185.39 | Current study |
| *Tadarida aegyptiaca/Mops condylurus* | Northern Limpopo, South Africa | Game Farm | Fur | Al Semi Quantitative |  | 102.62 |  | 51.44 | 558.06 | Current study |
| *Mops condylurus* | Northern Limpopo, South Africa | Diamond Mining | Fur | As |  | 0.72 |  | 0.37 | 1.20 | Current study |
| *Tadarida aegyptiaca/Mops condylurus* | Northern Limpopo, South Africa | Game Farm | Fur | As |  | 0.26 |  | 0.18 | 0.32 | Current study |
| *Hypsugo savii/Nyctalus leisleri /Pipistrellus pipistrellus/P.pygmaeus* | North and central Portugal | Wind farm | Fur | As |  | 0.22 |  | 0.05 | 1.91 | Mina et al. 2019* |
| *Myotis myotis* | Sicily | Petrochemical Plant | Fur | As | 0.16 | 0.11 |  | <0,01 | 1.02 | Ferrante et al. 2018* |
| *Myotis myotis* | Sicily | Pipistrelli Cave | Fur | As | 0.17 | 0.14 |  | <0,01 | 0.55 | Ferrante et al. 2018* |
| *Neoromicia nana* | Kwa-Zulu Natal, South Africa | Verlam wastewater treatment works | Fur | As | 0.38 |  |  | 0.15 | 0.64 | Hill et al. 2017* |
| *Neoromicia nana* | Kwa-Zulu Natal, South Africa | Umbilo wastewater treament works | Fur | As | 0.20 |  |  | 0.10 | 0.49 | Hill et al. 2017* |
| *Neoromicia nana* | Kwa-Zulu Natal, South Africa | Reference Sites | Fur | As | 0.10 |  |  | 0.01 | 0.29 | Hill et al. 2017* |
| *Mops condylurus* | Northern Limpopo, South Africa | Diamond Mining | Fur | B |  | 7.44 |  | 6.79 | 18.90 | Current study |
| *Tadarida aegyptiaca/Mops condylurus* | Northern Limpopo, South Africa | Game Farm | Fur | B |  | 3.38 |  | 1.64 | 3.91 | Current study |
| *Tadarida teniotis (lactant)* | North America | Across range | Fur | Ba | 0.005 |  |  |  |  | Andreani et al. 2019 |
| *Mops condylurus* | Northern Limpopo, South Africa | Diamond Mining | Fur | Ba |  | 0.06 |  | 0.06 | 1.51 | Current study |
| *Tadarida aegyptiaca/Mops condylurus* | Northern Limpopo, South Africa | Game Farm | Fur | Ba |  | 0.06 |  | 0.06 | 2.26 | Current study |
| *Mops condylurus* | Northern Limpopo, South Africa | Diamond Mining | Fur | Ca |  | 0.42 |  | 0.32 | 0.83 | Current study |
| *Tadarida aegyptiaca/Mops condylurus* | Northern Limpopo, South Africa | Game Farm | Fur | Ca |  | 0.38 |  | 0.27 | 0.82 | Current study |
| *Pipistrellus sp.* | England & Wales | Mixed | Fur | Cd | 0.004 - 212,00 | 0.1 |  |  |  | Hernout et al. 2016a |
| *Myotis bechsteinii* | Middle Hesse, Germany | Mixed: Urban, agriculture, forest | Fur | Cd |  | 0.05 |  | 0.0001 | 0.15 | Flache et al. 2015* |
| *Myotis daubentonii* | Middle Hesse, Germany | Mixed: Urban, agriculture, forest | Fur | Cd |  | 0.005 |  | 0.0001 | 0.09 | Flache et al. 2015* |
| *Myotis myotis* | Middle Hesse, Germany | Mixed: Urban, agriculture, forest | Fur | Cd |  | 0.07 |  | 0.02 | 0.36 | Flache et al. 2015* |
| *Pipistrellus pipistrellus* | Middle Hesse, Germany | Mixed: Urban, agriculture, forest | Fur | Cd |  | 0.20 |  | 0.0001 | 61.25 | Flache et al. 2015* |
| *Myotis bechsteinii* | Central Hesse, Germany | Mixed: Urban, agriculture, forest | Fur | Cd |  | 0.05 |  | 0.00 | 0.15 | Flache et al. 2018* |
| *Myotis nattereri* | Central Hesse, Germany | Mixed: Urban, agriculture, forest | Fur | Cd |  | 0.05 |  | 0.00 | 0.10 | Flache et al. 2018* |
| *Plecotus auritus* | Central Hesse, Germany | Mixed: Urban, agriculture, forest | Fur | Cd |  | 0.05 |  | 0.00 | 0.13 | Flache et al. 2018* |
| *Mops condylurus* | Northern Limpopo, South Africa | Diamond Mining | Fur | Cd |  | 0.09 |  | 0.03 | 0.17 | Current study |
| *Tadarida aegyptiaca/Mops condylurus* | Northern Limpopo, South Africa | Game Farm | Fur | Cd |  | 0.02 |  | 0.02 | 0.10 | Current study |
| *Hypsugo savii/Nyctalus leisleri /Pipistrellus pipistrellus/P.pygmaeus* | North and central Portugal | Wind farm | Fur | Cd |  | 0.02 |  | 0.01 | 0.14 | Mina et al. 2019* |
| *Myotis myotis* | Sicily | Petrochemical Plant | Fur | Cd | 0.004 | 0.004 |  | <0,01 | 0.02 | Ferrante et al. 2018* |
| *Myotis myotis* | Sicily | Pipistrelli Cave | Fur | Cd | 0.004 | 0.004 |  | <0,01 | 0.01 | Ferrante et al. 2018* |
| *Neoromicia nana* | Kwa-Zulu Natal, South Africa | Verlam wastewater treatment works | Fur | Cd | 0.02 |  |  | 0.004 | 0.07 | Hill et al. 2017* |
| *Neoromicia nana* | Kwa-Zulu Natal, South Africa | Umbilo wastewater treament works | Fur | Cd | 0.03 |  |  | 0.004 | 0.20 | Hill et al. 2017* |
| *Neoromicia nana* | Kwa-Zulu Natal, South Africa | Reference Sites | Fur | Cd | 0.01 |  |  | 0.005 | 0.04 | Hill et al. 2017* |
| *Lasiurus borealis* | North America | Across range | Fur | Cd | 0.0003 |  |  |  |  | Wieringa et al. 2020 |
| *Mops condylurus* | Northern Limpopo, South Africa | Diamond Mining | Fur | Co |  | 0.13 |  | 0.10 | 0.20 | Current study |
| *Tadarida aegyptiaca/Mops condylurus* | Northern Limpopo, South Africa | Game Farm | Fur | Co |  | 0.24 |  | 0.17 | 0.67 | Current study |
| *Hypsugo savii/Nyctalus leisleri /Pipistrellus pipistrellus/P.pygmaeus* | North and central Portugal | Wind farm | Fur | Co |  | 0.09 |  | 0.02 | 1.96 | Mina et al. 2019* |
| *Myotis myotis* | Sicily | Petrochemical Plant | Fur | Co | 0.01 | 0.01 |  | 0.003 | 0.02 | Ferrante et al. 2018* |
| *Myotis myotis* | Sicily | Pipistrelli Cave | Fur | Co | 0.02 | 0.02 |  | 0.003 | 0.22 | Ferrante et al. 2018* |
| *Mops condylurus* | Northern Limpopo, South Africa | Diamond Mining | Fur | Cr |  | 0.99 |  | 0.55 | 1.47 | Current study |
| *Tadarida aegyptiaca/Mops condylurus* | Northern Limpopo, South Africa | Game Farm | Fur | Cr |  | 1.07 |  | 0.37 | 2.71 | Current study |
| *Hypsugo savii/Nyctalus leisleri /Pipistrellus pipistrellus/P.pygmaeus* | North and central Portugal | Wind farm | Fur | Cr |  | 0.63 |  | 0.21 | 2.17 | Mina et al. 2019* |
| *Myotis myotis* | Sicily | Petrochemical Plant | Fur | Cr | 0.26 | 0.22 |  | 0.07 | 1.27 | Ferrante et al. 2018* |
| *Myotis myotis* | Sicily | Pipistrelli Cave | Fur | Cr | 0.29 | 0.25 |  | 0.12 | 0.80 | Ferrante et al. 2018* |
| *Neoromicia nana* | Kwa-Zulu Natal, South Africa | Verlam wastewater treatment works | Fur | Cr | 0.14 |  |  | 0.01 | 0.84 | Hill et al. 2017* |
| *Neoromicia nana* | Kwa-Zulu Natal, South Africa | Umbilo wastewater treament works | Fur | Cr | 0.06 |  |  | 0.01 | 0.27 | Hill et al. 2017* |
| *Neoromicia nana* | Kwa-Zulu Natal, South Africa | Reference Sites | Fur | Cr | 0.06 |  |  | 0.01 | 0.14 | Hill et al. 2017* |
| *Lasiurus borealis* | North America | Across range | Fur | Cs | 0.001 |  |  |  |  | Wieringa et al. 2020 |
| *Pipistrellus sp.* | England & Wales | Mixed | Fur | Cu | 2.20 - 103,00 | 14.96 |  |  |  | Hernout et al. 2016a |
| *Myotis bechsteinii* | Middle Hesse, Germany | Mixed: Urban, agriculture, forest | Fur | Cu |  | 7.80 |  | 1.43 | 31.25 | Flache et al. 2015* |
| *Myotis daubentonii* | Middle Hesse, Germany | Mixed: Urban, agriculture, forest | Fur | Cu |  | 17.55 |  | 7.43 | 121.50 | Flache et al. 2015* |
| *Myotis myotis* | Middle Hesse, Germany | Mixed: Urban, agriculture, forest | Fur | Cu |  | 5.23 |  | 0.78 | 23.70 | Flache et al. 2015* |
| *Pipistrellus pipistrellus* | Middle Hesse, Germany | Mixed: Urban, agriculture, forest | Fur | Cu |  | 9.05 |  | 3.65 | 44.00 | Flache et al. 2015* |
| *Myotis bechsteinii* | Central Hesse, Germany | Mixed: Urban, agriculture, forest | Fur | Cu |  | 7.50 |  | 1.25 | 17.50 | Flache et al. 2018* |
| *Myotis nattereri* | Central Hesse, Germany | Mixed: Urban, agriculture, forest | Fur | Cu |  | 18.75 |  | 5.50 | 22.75 | Flache et al. 2018* |
| *Plecotus auritus* | Central Hesse, Germany | Mixed: Urban, agriculture, forest | Fur | Cu |  | 6.50 |  | 0.00 | 26.00 | Flache et al. 2018* |
| *Mops condylurus* | Northern Limpopo, South Africa | Diamond Mining | Fur | Cu |  | 8.43 |  | 5.69 | 26.11 | Current study |
| *Tadarida aegyptiaca/Mops condylurus* | Northern Limpopo, South Africa | Game Farm | Fur | Cu |  | 6.09 |  | 5.34 | 17.41 | Current study |
| *Hypsugo savii/Nyctalus leisleri /Pipistrellus pipistrellus/P.pygmaeus* | North and central Portugal | Wind farm | Fur | Cu |  | 3.14 |  | 1.72 | 12.41 | Mina et al. 2019* |
| *Myotis myotis* | Sicily | Petrochemical Plant | Fur | Cu | 1.37 | 1.30 |  | 0.36 | 2.37 | Ferrante et al. 2018* |
| *Myotis myotis* | Sicily | Pipistrelli Cave | Fur | Cu | 1.94 | 1.84 |  | 0.95 | 3.13 | Ferrante et al. 2018* |
| *Lasiurus borealis* | North America | Across range | Fur | Cu | 0.006 |  |  |  |  | Wieringa et al. 2020 |
| *Neoromicia nana* | Kwa-Zulu Natal, South Africa | Verlam wastewater treatment works | Fur | Cu | 1.31 |  |  | 0.01 | 11.68 | Hill et al. 2017* |
| *Neoromicia nana* | Kwa-Zulu Natal, South Africa | Umbilo wastewater treament works | Fur | Cu | 1.48 |  |  | 0.01 | 8.64 | Hill et al. 2017* |
| *Neoromicia nana* | Kwa-Zulu Natal, South Africa | Reference Sites | Fur | Cu | 9.42 |  |  | 0.01 | 47.07 | Hill et al. 2017* |
| *Mops condylurus* | Northern Limpopo, South Africa | Diamond Mining | Fur | Fe |  | 101.41 |  | 67.54 | 181.25 | Current study |
| *Tadarida aegyptiaca/Mops condylurus* | Northern Limpopo, South Africa | Game Farm | Fur | Fe |  | 147.64 |  | 79.05 | 814.00 | Current study |
| *Myotis lucifugus* | Lafleche-1 Cave, Quebec, Canada | Cave | Fur | Fe | 220.00 |  |  |  |  | Hickey et al. 2001 |
| *Myotis lucifugus* | Lafleche 2 Cave, Quebec, Canada | Cave | Fur | Fe | 220.00 |  |  |  |  | Hickey et al. 2001 |
| *Myotis lucifugus* | Tyendinaga, Ontario, Canada | Mixed urban | Fur | Fe | 79.00 |  |  |  |  | Hickey et al. 2001 |
| *Myotis lucifugus* | Fly Creek, Ontario, Canada | Mixed urban | Fur | Fe | 72.00 |  |  |  |  | Hickey et al. 2001 |
| *Eptesicus fuscus* | Cornwall, Ontario, Canada | Mixed urban | Fur | Fe | 100.00 |  |  |  |  | Hickey et al. 2001 |
| *Lasiurus borealis* | North America | Across range | Fur | Fe | 0.065 |  |  |  |  | Wieringa et al. 2020 |
| *Neoromicia nana* | Kwa-Zulu Natal, South Africa | Verlam wastewater treatment works | Fur | Fe | 14.87 |  |  | 5.63 | 33.52 | Hill et al. 2017* |
| *Neoromicia nana* | Kwa-Zulu Natal, South Africa | Umbilo wastewater treament works | Fur | Fe | 21.14 |  |  | 5.95 | 42.63 | Hill et al. 2017* |
| *Neoromicia nana* | Kwa-Zulu Natal, South Africa | Reference Sites | Fur | Fe | 15.32 |  |  | 6.16 | 31.33 | Hill et al. 2017* |
| *Bauerus dubiaquercus* | Belize | Gold minining & Agriculture | Fur | Hg | 1.05 |  |  |  |  | Becker et al. 2018 |
| *Eptesicus furinalis* | Belize | Gold minining & Agriculture | Fur | Hg | 6.46 |  |  |  |  | Becker et al. 2018 |
| *Molossus rufus* | Belize | Gold minining & Agriculture | Fur | Hg | 2.91 |  |  |  |  | Becker et al. 2018 |
| *Myotis elegans* | Belize | Gold minining & Agriculture | Fur | Hg | 4.39 |  |  |  |  | Becker et al. 2018 |
| *Pteronotus davyi* | Belize | Gold minining & Agriculture | Fur | Hg | 3.92 |  |  |  |  | Becker et al. 2018 |
| *Pteronotus mesoamericanus* | Belize | Gold minining & Agriculture | Fur | Hg | 10.19 |  |  |  |  | Becker et al. 2018 |
| *Rhogeessa aeneus* | Belize | Gold minining & Agriculture | Fur | Hg | 4.87 |  |  |  |  | Becker et al. 2018 |
| *Rhynchonycteris naso* | Belize | Gold minining & Agriculture | Fur | Hg | 24.85 |  |  |  |  | Becker et al. 2018 |
| *Saccopteryx bilineata* | Belize | Gold minining & Agriculture | Fur | Hg | 6.44 |  |  |  |  | Becker et al. 2018 |
| *Mops condylurus* | Northern Limpopo, South Africa | Diamond Mining | Fur | Hg |  | 1.27 |  | 0.58 | 1.95 | Current study |
| *Tadarida aegyptiaca/Mops condylurus* | Northern Limpopo, South Africa | Game Farm | Fur | Hg |  | 0.60 |  | 0.39 | 1.11 | Current study |
| *Myotis lucifugus* | Lafleche-1 Cave, Quebec, Canada | Cave | Fur | Hg | 2.00 |  |  |  |  | Hickey et al. 2001 |
| *Myotis lucifugus* | Lafleche 2 Cave, Quebec, Canada | Cave | Fur | Hg | 2.80 |  |  |  |  | Hickey et al. 2001 |
| *Myotis lucifugus* | Tyendinaga, Ontario, Canada | Mixed urban | Fur | Hg | 7.60 |  |  |  |  | Hickey et al. 2001 |
| *Myotis lucifugus* | Fly Creek, Ontario, Canada | Mixed urban | Fur | Hg | 3.90 |  |  |  |  | Hickey et al. 2001 |
| *Eptesicus fuscus* | Cornwall, Ontario, Canada | Mixed urban | Fur | Hg | 4.60 |  |  |  |  | Hickey et al. 2001 |
| *Myotis lucifugus* | Canada | - | Fur | Hg |  |  | 1.50 | 1.30 | 2.50 | Hickey et al. 2001 |
| *Myotis septentionalis* | Canada | - | Fur | Hg |  |  | 4.40 | -1.20 | 10.20 | Hickey et al. 2001 |
| *Eptesicus fuscus* | Canada | - | Fur | Hg |  |  | 1.50 | -5.00 | 15.40 | Hickey et al. 2001 |
| *Myotis leibii* | Canada | - | Fur | Hg |  |  | 5.30 | -61.00 | 76.20 | Hickey et al. 2001 |
| *Eptesicus furinalis* | Peru | Small-scale gold mining - Papya plantation | Fur | Hg | 1.25 |  |  | - | - | Carrasco-Rueda et al. 2020* |
| *Lophostoma silvicolum* | Peru | Small-scale gold mining - Papya plantation | Fur | Hg | 0.27 |  |  | - | - | Carrasco-Rueda et al. 2020* |
| *Micronycteris minuta* | Peru | Small-scale gold mining - Papya plantation | Fur | Hg | 0.35 |  |  | - | - | Carrasco-Rueda et al. 2020* |
| *Mimon crenulatum* | Peru | Small-scale gold mining - Papya plantation | Fur | Hg | 0.73 |  |  | - | - | Carrasco-Rueda et al. 2020* |
| *Tonatia suarophila* | Peru | Small-scale gold mining - Papya plantation | Fur | Hg | 0.68 |  |  | - | - | Carrasco-Rueda et al. 2020* |
| *Trinycteris nicefori* | Peru | Small-scale gold mining - Papya plantation | Fur | Hg | 0.01 |  |  | - | - | Carrasco-Rueda et al. 2020* |
| *Eptesicus brasiliensis* | Peru | Small-scale gold mining - Cattle pasture | Fur | Hg | 1.56 |  |  | - | - | Carrasco-Rueda et al. 2020* |
| *Eptesicus furinalis* | Peru | Small-scale gold mining - Cattle pasture | Fur | Hg | 1.15 |  |  | - | - | Carrasco-Rueda et al. 2020* |
| *Lophostoma silvicolum* | Peru | Small-scale gold mining - Cattle pasture | Fur | Hg | 0.01 |  |  | - | - | Carrasco-Rueda et al. 2020* |
| *Molossus molossus* | Peru | Small-scale gold mining - Cattle pasture | Fur | Hg | 0.83 |  |  | - | - | Carrasco-Rueda et al. 2020* |
| *Molossus cf. rufuc* | Peru | Small-scale gold mining - Cattle pasture | Fur | Hg | 0.42 |  |  | - | - | Carrasco-Rueda et al. 2020* |
| *Saccopteryx bilineata* | Peru | Small-scale gold mining - Cattle pasture | Fur | Hg | 1.96 |  |  | - | - | Carrasco-Rueda et al. 2020* |
| *Myotis myotis* | Sicily | Petrochemical Plant | Fur | Hg | 0.59 | 0.46 |  | 0.16 | 2.35 | Ferrante et al. 2018* |
| *Myotis myotis* | Sicily | Pipistrelli Cave | Fur | Hg | 0.69 | 0.61 |  | 0.04 | 2.57 | Ferrante et al. 2018* |
| *Lasiurus borealis* | North America | Across range | Fur | Hg | 0.005 |  |  | - | - | Wieringa et al. 2020 |
| *Perimyotis subflavus* | Northeast United States | Point source and non-point source | Fur | Hg | 0.74 | 0.42 |  | - | 2.75 | Yates et al. 2014 |
| *Myotis lucifugus* | Northeast United States | Point source and non-point source | Fur | Hg | 0.28 | 0.04 |  | - | 3.76 | Yates et al. 2014 |
| *Myotis septentionalis* | Northeast United States | Point source and non-point source | Fur | Hg | 0.6 | 0.12 |  | - | 3.7 | Yates et al. 2014 |
| *Myotis grisescens* | Northeast United States | Point source and non-point source | Fur | Hg | 0.12 | 0.02 |  | - | 0.46 | Yates et al. 2014 |
| *Eptesicus fuscus* | Northeast United States | Point source and non-point source | Fur | Hg | 0.1 | 0.06 |  | - | 0.89 | Yates et al. 2014 |
| *Myotis sodalis* | Northeast United States | Point source and non-point source | Fur | Hg | - | - |  | - | - | Yates et al. 2014 |
| *Myotis leibii* | Northeast United States | Point source and non-point source | Fur | Hg | - | - |  | - | - | Yates et al. 2014 |
| *Lasionycteris noctivagans* | Northeast United States | Point source and non-point source | Fur | Hg | - | - |  | - | - | Yates et al. 2014 |
| *Lasiurus borealis* | Northeast United States | Point source and non-point source | Fur | Hg | 0.05 | 0.03 |  | - | 0.22 | Yates et al. 2014 |
| *Lasiurus cinereus* | Northeast United States | Point source and non-point source | Fur | Hg | 0.02 | 0.01 |  | - | 0.03 | Yates et al. 2014 |
| *Myotis lucifugus* | Moscow, Russia | Uncontaminated | Fur | Hg | 3.30 |  |  | 0.60 | 14.90 | Karouna-Renier et al. 2014 |
| *Myotis lucifugus* | Northwestern Virginia, USA | Contaminated (Grottoes & Mt. Sydney | Fur | Hg | 118.40 |  |  | 2.00 | 707.60 | Karouna-Renier et al. 2014 |
| *Myotis lucifugus* | Northwestern Virginia, USA | Contaminated (Grottoes) | Fur | Hg | 189.10 |  |  | 2.90 | 707.60 | Karouna-Renier et al. 2014 |
| *Myotis lucifugus* | Northwestern Virginia, USA | Contaminated (Mt. Sydney | Fur | Hg | 28.90 |  |  | 2.40 | 320.80 | Karouna-Renier et al. 2014 |
| *Mops condylurus* | Northern Limpopo, South Africa | Diamond Mining | Fur | K |  | 3.31 |  | 2.54 | 7.30 | Current study |
| *Tadarida aegyptiaca/Mops condylurus* | Northern Limpopo, South Africa | Game Farm | Fur | K |  | 1.33 |  | 0.77 | 2.14 | Current study |
| *Lasiurus borealis* | North America | Across range | Fur | Mg | 0.052 |  |  |  |  | Wieringa et al. 2020 |
| *Myotis bechsteinii* | Middle Hesse, Germany | Mixed: Urban, agriculture, forest | Fur | Mn | 8.48 |  |  | 0.002 | 18.78 | Flache et al. 2015* |
| *Myotis daubentonii* | Middle Hesse, Germany | Mixed: Urban, agriculture, forest | Fur | Mn | 5.88 |  |  | 0.002 | 12.38 | Flache et al. 2015* |
| *Myotis myotis* | Middle Hesse, Germany | Mixed: Urban, agriculture, forest | Fur | Mn | 16.38 |  |  | 7.98 | 35.25 | Flache et al. 2015* |
| *Pipistrellus pipistrellus* | Middle Hesse, Germany | Mixed: Urban, agriculture, forest | Fur | Mn | 6.70 |  |  | 0.31 | 23.20 | Flache et al. 2015* |
| *Myotis bechsteinii* | Central Hesse, Germany | Mixed: Urban, agriculture, forest | Fur | Mn |  | 7.50 |  | 0.00 | 19.00 | Flache et al. 2018* |
| *Myotis nattereri* | Central Hesse, Germany | Mixed: Urban, agriculture, forest | Fur | Mn |  | 0 |  | 0.00 | 3.00 | Flache et al. 2018* |
| *Plecotus auritus* | Central Hesse, Germany | Mixed: Urban, agriculture, forest | Fur | Mn |  | 10.25 |  | 0.00 | 10.88 | Flache et al. 2018* |
| *Mops condylurus* | Northern Limpopo, South Africa | Diamond Mining | Fur | Mn |  | 4.75 |  | 3.23 | 8.01 | Current study |
| *Tadarida aegyptiaca/Mops condylurus* | Northern Limpopo, South Africa | Game Farm | Fur | Mn |  | 13.33 |  | 6.19 | 32.61 | Current study |
| *Hypsugo savii/Nyctalus leisleri /Pipistrellus pipistrellus/P.pygmaeus* | North and central Portugal | Wind farm | Fur | Mn |  | 2.84 |  | 0.93 | 27.75 | Mina et al. 2019* |
| *Myotis myotis* | Sicily | Petrochemical Plant | Fur | Mn | 0.73 | 0.55 |  | 0.13 | 4.66 | Ferrante et al. 2018* |
| *Myotis myotis* | Sicily | Pipistrelli Cave | Fur | Mn | 0.85 | 0.73 |  | 0.13 | 2.78 | Ferrante et al. 2018* |
| *Lasiurus borealis* | North America | Across range | Fur | Mn | 0.001 |  |  |  |  | Wieringa et al. 2020 |
| *Neoromicia nana* | Kwa-Zulu Natal, South Africa | Verlam wastewater treatment works | Fur | Mn | 2.41 |  |  | 0.78 | 6.71 | Hill et al. 2017* |
| *Neoromicia nana* | Kwa-Zulu Natal, South Africa | Umbilo wastewater treament works | Fur | Mn | 3.00 |  |  | 0.79 | 7.83 | Hill et al. 2017* |
| *Neoromicia nana* | Kwa-Zulu Natal, South Africa | Reference Sites | Fur | Mn | 7.67 |  |  | 2.42 | 16.80 | Hill et al. 2017* |
| *Myotis bechsteinii* | Central Hesse, Germany | Mixed: Urban, agriculture, forest | Fur | Mo |  | 0.19 |  | 0.00 | 0.38 | Flache et al. 2018* |
| *Myotis nattereri* | Central Hesse, Germany | Mixed: Urban, agriculture, forest | Fur | Mo |  | 0.35 |  | 0.16 | 0.76 | Flache et al. 2018* |
| *Plecotus auritus* | Central Hesse, Germany | Mixed: Urban, agriculture, forest | Fur | Mo |  | 0.21 |  | 0.10 | 0.55 | Flache et al. 2018* |
| *Mops condylurus* | Northern Limpopo, South Africa | Diamond Mining | Fur | Mo |  | 0.85 |  | 0.59 | 0.92 | Current study |
| *Tadarida aegyptiaca/Mops condylurus* | Northern Limpopo, South Africa | Game Farm | Fur | Mo |  | 0.72 |  | 0.52 | 0.82 | Current study |
| *Lasiurus borealis* | North America | Across range | Fur | Mo | 0.002 |  |  |  |  | Wieringa et al. 2020 |
| *Mops condylurus* | Northern Limpopo, South Africa | Diamond Mining | Fur | Ni |  | 1.13 |  | 0.79 | 2.97 | Current study |
| *Tadarida aegyptiaca/Mops condylurus* | Northern Limpopo, South Africa | Game Farm | Fur | Ni |  | 1.38 |  | 0.77 | 2.58 | Current study |
| *Hypsugo savii/Nyctalus leisleri /Pipistrellus pipistrellus/P.pygmaeus* | North and central Portugal | Wind farm | Fur | Ni |  | 0.65 |  | 0.15 | 11.22 | Mina et al. 2019* |
| *Myotis myotis* | Sicily | Petrochemical Plant | Fur | Ni | 0.11 | 0.06 |  | <0,02 | 0.86 | Ferrante et al. 2018* |
| *Myotis myotis* | Sicily | Pipistrelli Cave | Fur | Ni | 0.14 | 0.11 |  | 0.01 | 0.77 | Ferrante et al. 2018* |
| *Lasiurus borealis* | North America | Across range | Fur | Ni | 0.002 |  |  |  |  | Wieringa et al. 2020 |
| *Pipistrellus pipistrellus* | England & Wales | Mixed | Fur | Pb | 286.000 |  |  |  |  | Hernout et al. 2016a |
| *Pipistrellus pygmaeus* | England & Wales | Mixed | Fur | Pb | 5558.000 |  |  |  |  | Hernout et al. 2016a |
| *Pipistrellus sp.* | England & Wales | Mixed | Fur | Pb | 0.045 - 20399,00 | 28.800 |  |  |  | Hernout et al. 2016a |
| *Myotis bechsteinii* | Middle Hesse, Germany | Mixed: Urban, agriculture, forest | Fur | Pb |  | 1.29 |  | 0.45 | 2.16 | Flache et al. 2015* |
| *Myotis daubentonii* | Middle Hesse, Germany | Mixed: Urban, agriculture, forest | Fur | Pb |  | 1.07 |  | 0.005 | 5.15 | Flache et al. 2015* |
| *Myotis myotis* | Middle Hesse, Germany | Mixed: Urban, agriculture, forest | Fur | Pb |  | 0.10 |  | 0.004 | 1.68 | Flache et al. 2015* |
| *Pipistrellus pipistrellus* | Middle Hesse, Germany | Mixed: Urban, agriculture, forest | Fur | Pb |  | 8.55 |  | 0.004 | 129.75 | Flache et al. 2015* |
| *Myotis bechsteinii* | Central Hesse, Germany | Mixed: Urban, agriculture, forest | Fur | Pb |  | 1.25 |  | 0.45 | 2.20 | Flache et al. 2018* |
| *Myotis nattereri* | Central Hesse, Germany | Mixed: Urban, agriculture, forest | Fur | Pb |  | 1.50 |  | 0.28 | 3.40 | Flache et al. 2018* |
| *Plecotus auritus* | Central Hesse, Germany | Mixed: Urban, agriculture, forest | Fur | Pb |  | 0.70 |  | 0.00 | 3.20 | Flache et al. 2018* |
| *Mops condylurus* | Northern Limpopo, South Africa | Diamond Mining | Fur | Pb |  | 0.450 |  | 0.16 | 0.94 | Current study |
| *Tadarida aegyptiaca/Mops condylurus* | Northern Limpopo, South Africa | Game Farm | Fur | Pb |  | 0.480 |  | 0.42 | 1.47 | Current study |
| *Hypsugo savii/Nyctalus leisleri /Pipistrellus pipistrellus/P.pygmaeus* | North and central Portugal | Wind farm | Fur | Pb |  | 0.63 |  | 0.30 | 14.39 | Mina et al. 2019* |
| *Myotis lucifugus* | Lafleche-1 Cave, Quebec, Canada | Cave | Fur | Pb | 2.500 |  |  |  |  | Hickey et al. 2001 |
| *Myotis lucifugus* | Lafleche 2 Cave, Quebec, Canada | Cave | Fur | Pb | 3.000 |  |  |  |  | Hickey et al. 2001 |
| *Myotis lucifugus* | Tyendinaga, Ontario, Canada | Mixed urban | Fur | Pb | 1.600 |  |  |  |  | Hickey et al. 2001 |
| *Myotis lucifugus* | Fly Creek, Ontario, Canada | Mixed urban | Fur | Pb | 6.200 |  |  |  |  | Hickey et al. 2001 |
| *Eptesicus fuscus* | Cornwall, Ontario, Canada | Mixed urban | Fur | Pb | 8.800 |  |  |  |  | Hickey et al. 2001 |
| *Myotis lucifugus* | Canada | - | Fur | Pb |  |  | ND-11.3 |  |  | Hickey et al. 2001 |
| *Myotis septentionalis* | Canada | - | Fur | Pb |  |  | - |  |  | Hickey et al. 2001 |
| *Eptesicus fuscus* | Canada | - | Fur | Pb |  |  | ND-6.1 |  |  | Hickey et al. 2001 |
| *Myotis leibii* | Canada | - | Fur | Pb |  |  | - |  |  | Hickey et al. 2001 |
| *Myotis myotis* | Sicily | Petrochemical Plant | Fur | Pb | 0.19 | 0.06 |  | 0.01 | 1.52 | Ferrante et al. 2018* |
| *Myotis myotis* | Sicily | Pipistrelli Cave | Fur | Pb | 0.11 | 0.07 |  | 0.01 | 0.43 | Ferrante et al. 2018* |
| *Neoromicia nana* | Kwa-Zulu Natal, South Africa | Verlam wastewater treatment works | Fur | Pb | 0.46 |  |  | 0.01 | 1.55 | Hill et al. 2017* |
| *Neoromicia nana* | Kwa-Zulu Natal, South Africa | Umbilo wastewater treament works | Fur | Pb | 0.39 |  |  | 0.01 | 1.74 | Hill et al. 2017* |
| *Neoromicia nana* | Kwa-Zulu Natal, South Africa | Reference Sites | Fur | Pb | 1.49 |  |  | 0.01 | 6.65 | Hill et al. 2017* |
| *Mops condylurus* | Northern Limpopo, South Africa | Diamond Mining | Fur | Rb |  | 1.260 |  | 1.11 | 2.96 | Current study |
| *Tadarida aegyptiaca/Mops condylurus* | Northern Limpopo, South Africa | Game Farm | Fur | Rb |  | 0.890 |  | 0.63 | 1.06 | Current study |
| *Lasiurus borealis* | North America | Across range | Fur | Rb | 0.001 |  |  |  |  | Wieringa et al. 2020 |
| *Mops condylurus* | Northern Limpopo, South Africa | Diamond Mining | Fur | Sb |  | 0.180 |  | 0.09 | 0.22 | Current study |
| *Tadarida aegyptiaca/Mops condylurus* | Northern Limpopo, South Africa | Game Farm | Fur | Sb |  | 0.040 |  | 0.02 | 0.07 | Current study |
| *Myotis myotis* | Sicily | Petrochemical Plant | Fur | Sb | 0.01 | 0.01 |  | <0,01 | 0.04 | Ferrante et al. 2018* |
| *Myotis myotis* | Sicily | Pipistrelli Cave | Fur | Sb | 0.02 | 0.01 |  | <0,01 | 0.05 | Ferrante et al. 2018* |
| *Mops condylurus* | Northern Limpopo, South Africa | Diamond Mining | Fur | Se |  | 5.480 |  | 3.65 | 8.21 | Current study |
| *Tadarida aegyptiaca/Mops condylurus* | Northern Limpopo, South Africa | Game Farm | Fur | Se |  | 4.030 |  | 1.95 | 6.40 | Current study |
| *Hypsugo savii/Nyctalus leisleri /Pipistrellus pipistrellus/P.pygmaeus* | North and central Portugal | Wind farm | Fur | Se |  | 0.86 |  | 0.24 | 5.48 | Mina et al. 2019* |
| *Myotis lucifugus* | Lafleche-1 Cave, Quebec, Canada | Cave | Fur | Se | 17.000 |  |  |  |  | Hickey et al. 2001 |
| *Myotis lucifugus* | Lafleche 2 Cave, Quebec, Canada | Cave | Fur | Se | 13.000 |  |  |  |  | Hickey et al. 2001 |
| *Myotis lucifugus* | Tyendinaga, Ontario, Canada | Mixed urban | Fur | Se | 22.000 |  |  |  |  | Hickey et al. 2001 |
| *Myotis lucifugus* | Fly Creek, Ontario, Canada | Mixed urban | Fur | Se | 69.000 |  |  |  |  | Hickey et al. 2001 |
| *Eptesicus fuscus* | Cornwall, Ontario, Canada | Mixed urban | Fur | Se | 9.500 |  |  |  |  | Hickey et al. 2001 |
| *Myotis lucifugus* | Canada | - | Fur | Se |  |  | ND-26.90 |  |  | Hickey et al. 2001 |
| *Myotis septentionalis* | Canada | - | Fur | Se |  |  | - |  |  | Hickey et al. 2001 |
| *Eptesicus fuscus* | Canada | - | Fur | Se |  |  | - |  |  | Hickey et al. 2001 |
| *Myotis leibii* | Canada | - | Fur | Se |  |  | - |  |  | Hickey et al. 2001 |
| *Myotis myotis* | Sicily | Petrochemical Plant | Fur | Se | 0.20 | 0.19 |  | 0.08 | 0.42 | Ferrante et al. 2018* |
| *Myotis myotis* | Sicily | Pipistrelli Cave | Fur | Se | 0.27 | 0.27 |  | 0.13 | 0.43 | Ferrante et al. 2018* |
| *Mops condylurus* | Northern Limpopo, South Africa | Diamond Mining | Fur | Sn |  | 0.090 |  | 0.06 | 0.18 | Current study |
| *Tadarida aegyptiaca/Mops condylurus* | Northern Limpopo, South Africa | Game Farm | Fur | Sn |  | 0.060 |  | 0.03 | 0.16 | Current study |
| *Lasiurus borealis* | North America | Across range | Fur | Sn | 0.004 |  |  |  |  | Wieringa et al. 2020 |
| *Mops condylurus* | Northern Limpopo, South Africa | Diamond Mining | Fur | Sr |  | 1.440 |  | 1.02 | 1.85 | Current study |
| *Tadarida aegyptiaca/Mops condylurus* | Northern Limpopo, South Africa | Game Farm | Fur | Sr |  | 1.710 |  | 1.37 | 3.65 | Current study |
| *Mops condylurus* | Northern Limpopo, South Africa | Diamond Mining | Fur | V |  | 0.28 |  | 0.24 | 0.40 | Current study |
| *Tadarida aegyptiaca/Mops condylurus* | Northern Limpopo, South Africa | Game Farm | Fur | V |  | 0.52 |  | 0.34 | 1.24 | Current study |
| *Myotis myotis* | Sicily | Petrochemical Plant | Fur | V | 0.07 | 0.07 |  | <0,01 | 0.18 | Ferrante et al. 2018* |
| *Myotis myotis* | Sicily | Pipistrelli Cave | Fur | V | 0.11 | 0.11 |  | 0.03 | 0.29 | Ferrante et al. 2018* |
| *Lasiurus borealis* | North America | Across range | Fur | Y | 0.000 |  |  |  |  | Wieringa et al. 2020 |
| *Pipistrellus sp.* | England & Wales | Mixed | Fur | Zn | 11.8 - 578,00 | 72.970 |  |  |  | Hernout et al. 2016a |
| *Myotis bechsteinii* | Middle Hesse, Germany | Mixed: Urban, agriculture, forest | Fur | Zn |  | 25.75 |  | 13.25 | 36.50 | Flache et al. 2015* |
| *Myotis daubentonii* | Middle Hesse, Germany | Mixed: Urban, agriculture, forest | Fur | Zn |  | 32.25 |  | 23.38 | 35.50 | Flache et al. 2015* |
| *Myotis myotis* | Middle Hesse, Germany | Mixed: Urban, agriculture, forest | Fur | Zn |  | 22.13 |  | 15.33 | 29.75 | Flache et al. 2015* |
| *Pipistrellus pipistrellus* | Middle Hesse, Germany | Mixed: Urban, agriculture, forest | Fur | Zn |  | 95.75 |  | 70.50 | 288.75 | Flache et al. 2015* |
| *Myotis bechsteinii* | Central Hesse, Germany | Mixed: Urban, agriculture, forest | Fur | Zn |  | 26.50 |  | 14.00 | 36.00 | Flache et al. 2018* |
| *Myotis nattereri* | Central Hesse, Germany | Mixed: Urban, agriculture, forest | Fur | Zn |  | 23.44 |  | 14.00 | 31.69 | Flache et al. 2018* |
| *Plecotus auritus* | Central Hesse, Germany | Mixed: Urban, agriculture, forest | Fur | Zn |  | 32.75 |  | 24.94 | 43.70 | Flache et al. 2018* |
| *Mops condylurus* | Northern Limpopo, South Africa | Diamond Mining | Fur | Zn |  | 147.09 |  | 117.80 | 346.41 | Current study |
| *Tadarida aegyptiaca/Mops condylurus* | Northern Limpopo, South Africa | Game Farm | Fur | Zn |  | 97.70 |  | 37.78 | 241.87 | Current study |
| *Hypsugo savii/Nyctalus leisleri /Pipistrellus pipistrellus/P.pygmaeus* | North and central Portugal | Wind farm | Fur | Zn |  | 59.83 |  | 37.30 | 206.79 | Mina et al. 2019* |
| *Myotis lucifugus* | Lafleche-1 Cave, Quebec, Canada | Cave | Fur | Zn | 140.000 |  |  |  |  | Hickey et al. 2001 |
| *Myotis lucifugus* | Lafleche 2 Cave, Quebec, Canada | Cave | Fur | Zn | 200.000 |  |  |  |  | Hickey et al. 2001 |
| *Myotis lucifugus* | Tyendinaga, Ontario, Canada | Mixed urban | Fur | Zn | 130.000 |  |  |  |  | Hickey et al. 2001 |
| *Myotis lucifugus* | Fly Creek, Ontario, Canada | Mixed urban | Fur | Zn | 130.000 |  |  |  |  | Hickey et al. 2001 |
| *Eptesicus fuscus* | Cornwall, Ontario, Canada | Mixed urban | Fur | Zn | 160.000 |  |  |  |  | Hickey et al. 2001 |
| *Myotis lucifugus* | Canada | - | Fur | Zn |  |  | 101.400 | 94.900 | 110.100 | Hickey et al. 2001 |
| *Myotis septentionalis* | Canada | - | Fur | Zn |  |  | 107.600 | 94.400 | 121.600 | Hickey et al. 2001 |
| *Eptesicus fuscus* | Canada | - | Fur | Zn |  |  | 101.300 | 105.100 | 154.800 | Hickey et al. 2001 |
| *Myotis leibii* | Canada | - | Fur | Zn |  |  | 314.600 | -4513.00 | 5523.00 | Hickey et al. 2001 |
| *Neoromicia nana* | Kwa-Zulu Natal, South Africa | Verlam wastewater treatment works | Fur | Zn | 74.68 |  |  | 61.81 | 97.84 | Hill et al. 2017* |
| *Neoromicia nana* | Kwa-Zulu Natal, South Africa | Umbilo wastewater treament works | Fur | Zn | 77.15 |  |  | 51.65 | 138.60 | Hill et al. 2017* |
| *Neoromicia nana* | Kwa-Zulu Natal, South Africa | Reference Sites | Fur | Zn | 63.95 |  |  | 40.48 | 89.49 | Hill et al. 2017* |
| *Tadarida teniotis/Miniopterus schreibersii* | Italy | Mixed | Skin-fur | Al | 306.00 |  |  |  |  | Andreani et al. 2019 |
| *Tadarida teniotis/Miniopterus schreibersii* | Italy | Mixed | Skin-fur | As | 0.29 |  |  |  |  | Andreani et al. 2019 |
| *Tadarida teniotis/Miniopterus schreibersii* | Italy | Mixed | Skin-fur | Ba | 30.00 |  |  |  |  | Andreani et al. 2019 |
| *Tadarida teniotis/Miniopterus schreibersii* | Italy | Mixed | Skin-fur | Cd | 0.04 |  |  |  |  | Andreani et al. 2019 |
| *Tadarida teniotis/Miniopterus schreibersii* | Italy | Mixed | Skin-fur | Hg | 0.065 |  |  |  |  | Andreani et al. 2019 |
| *Tadarida teniotis/Miniopterus schreibersii* | Italy | Mixed | Skin-fur | Pb | 36.900 |  |  |  |  | Andreani et al. 2019 |
| *Tadarida teniotis/Miniopterus schreibersii* | Italy | Mixed | Skin-fur | Sb | 1.980 |  |  |  |  | Andreani et al. 2019 |
| *Tadarida teniotis/Miniopterus schreibersii* | Italy | Mixed | Skin-fur | Sr | 9.140 |  |  |  |  | Andreani et al. 2019 |
| *Tadarida teniotis/Miniopterus schreibersii* | Italy | Mixed | Skin-fur | Th | 0.273 |  |  |  |  | Andreani et al. 2019 |
| *Tadarida teniotis/Miniopterus schreibersii* | Italy | Mixed | Skin-fur | Tl | 0.072 |  |  |  |  | Andreani et al. 2019 |
| *Tadarida teniotis/Miniopterus schreibersii* | Italy | Mixed | Whole animal | Al | 47.95 |  |  |  |  | Andreani et al. 2019 |
| *Miniopterus schreibersii* | Italy | African Quarter of Rome | Whole animal | Al | 36.90 |  |  |  |  | Andreani et al. 2019 |
| *Tadarida teniotis/Miniopterus schreibersii* | Italy | Mixed | Whole animal | As | 0.05 |  |  |  |  | Andreani et al. 2019 |
| *Miniopterus schreibersii* | Italy | African Quarter of Rome | Whole animal | As | 0.08 |  |  |  |  | Andreani et al. 2019 |
| *Tadarida teniotis/Miniopterus schreibersii* | Italy | Mixed | Whole animal | Ba | 5.75 |  |  |  |  | Andreani et al. 2019 |
| *Miniopterus schreibersii* | Italy | African Quarter of Rome | Whole animal | Ba | 1.38 |  |  |  |  | Andreani et al. 2019 |
| *Tadarida teniotis/Miniopterus schreibersii* | Italy | Mixed | Whole animal | Cd | 0.01 |  |  |  |  | Andreani et al. 2019 |
| *Miniopterus schreibersii* | Italy | African Quarter of Rome | Whole animal | Cd | 0.04 |  |  |  |  | Andreani et al. 2019 |
| *Tadarida teniotis/Miniopterus schreibersii* | Italy | Mixed | Whole animal | Hg | 0.01 |  |  |  |  | Andreani et al. 2019 |
| *Miniopterus schreibersii* | Italy | Urban | Whole animal | Hg | 0.08 |  |  |  |  | Andreani et al. 2019 |
| *Tadarida teniotis/Miniopterus schreibersii* | Italy | Mixed | Whole animal | Pb | 39.380 |  |  |  |  | Andreani et al. 2019 |
| *Miniopterus schreibersii* | Italy | Urban | Whole animal | Pb | 0.310 |  |  |  |  | Andreani et al. 2019 |
| *Tadarida teniotis/Miniopterus schreibersii* | Italy | Mixed | Whole animal | Sb | 0.078 |  |  |  |  | Andreani et al. 2019 |
| *Miniopterus schreibersii* | Italy | Urban | Whole animal | Sb | <LOQ |  |  |  |  | Andreani et al. 2019 |
| *Tadarida teniotis/Miniopterus schreibersii* | Italy | Mixed | Whole animal | Sr | 9.800 |  |  |  |  | Andreani et al. 2019 |
| *Miniopterus schreibersii* | Italy | Urban | Whole animal | Sr | 28.200 |  |  |  |  | Andreani et al. 2019 |
